# IFACEwat: the interfacial water-implemented re-ranking algorithm to improve the discrimination of near native structures for protein rigid docking

**DOI:** 10.1186/1471-2105-15-S16-S9

**Published:** 2014-12-08

**Authors:** Chinh Tran-To Su, Thuy-Diem Nguyen, Jie Zheng, Chee-Keong Kwoh

**Affiliations:** 1 Bioinformatics Research Center, School of Computer Engineering, Nanyang Technological University, Singapore 639798; 2 Parallel and Distributed Computing Centre, School of Computer Engineering, Nanyang Technological University, Singapore 639798; 3 Genome Institute of Singapore, Agency for Science, Technology, and Research (A*STAR), Biopolis, Singapore 138672

## Abstract

**Background:**

Protein-protein docking is an *in silico *method to predict the formation of protein complexes. Due to limited computational resources, the protein-protein docking approach has been developed under the assumption of rigid docking, in which one of the two protein partners remains rigid during the protein associations and water contribution is ignored or implicitly presented. Despite obtaining a number of acceptable complex predictions, it seems to-date that most initial rigid docking algorithms still find it difficult or even fail to discriminate successfully the correct predictions from the other incorrect or false positive ones. To improve the rigid docking results, re-ranking is one of the effective methods that help re-locate the correct predictions in top high ranks, discriminating them from the other incorrect ones.

In this paper, we propose a new re-ranking technique using a new energy-based scoring function, namely IFACEwat - a combined Interface Atomic Contact Energy (IFACE) and water effect. The IFACEwat aims to further improve the discrimination of the near-native structures of the initial rigid docking algorithm ZDOCK3.0.2. Unlike other re-ranking techniques, the IFACEwat explicitly implements interfacial water into the protein interfaces to account for the water-mediated contacts during the protein interactions.

**Results:**

Our results showed that the IFACEwat increased both the numbers of the near-native structures and improved their ranks as compared to the initial rigid docking ZDOCK3.0.2. In fact, the IFACEwat achieved a success rate of 83.8% for Antigen/Antibody complexes, which is 10% better than ZDOCK3.0.2. As compared to another re-ranking technique ZRANK, the IFACEwat obtains success rates of 92.3% (8% better) and 90% (5% better) respectively for medium and difficult cases. When comparing with the latest published re-ranking method F^2^Dock, the IFACEwat performed equivalently well or even better for several Antigen/Antibody complexes.

**Conclusions:**

With the inclusion of interfacial water, the IFACEwat improves mostly results of the initial rigid docking, especially for Antigen/Antibody complexes. The improvement is achieved by explicitly taking into account the contribution of water during the protein interactions, which was ignored or not fully presented by the initial rigid docking and other re-ranking techniques. In addition, the IFACEwat maintains sufficient computational efficiency of the initial docking algorithm, yet improves the ranks as well as the number of the near native structures found. As our implementation so far targeted to improve the results of ZDOCK3.0.2, and particularly for the Antigen/Antibody complexes, it is expected in the near future that more implementations will be conducted to be applicable for other initial rigid docking algorithms.

## Background

Protein-protein interactions have been well studied by both lab experiments and computational simulations [1]. Understanding protein interactions is crucial for designing drugs and finding drug targets. While knowledge of protein interactions and their molecular pathways have been discovered experimentally, limited information about structures of the known protein complexes could be elucidated. In addition, due to the transient or obligatory associations, not every protein complex could be experimentally crystalized. Therefore predicting the complex formation using *in silico *method, e.g. protein-protein docking, has become an important complement with the *in vitro *studies in investigating protein-protein interactions.

Due to the compromise of protein flexibility against limited computational resources, most current protein docking algorithms are driven under the assumption of rigid docking, i.e. one of the protein partners remains rigid during the complex associations [2-11]. Hence, results of the rigid docking often require further refinement to obtain optimal structures of the protein complexes. However, this refinement stage is computationally intensive [12].

Although the rigid docking has successfully predicted formations of many protein complexes, it often fails if the proteins undergo conformational changes (e.g. Antigen/Antibody complexes) or their interactions are influenced by the solvent [13]. In fact, rigid docking results contain high false positive rates caused by a failure to locate the correct predictions from the other incorrect ones. Therefore, if the refinement is a crucial step that every protein docking algorithm needs to perform, it is important to improve the number of correct predictions while limiting the number of false positives that need to be refined in order to achieve better computational efficiency.

Re-ranking technique used in protein docking is an effective approach to discriminating the correct predictions from the others [14-17] by re-locating them in the top higher ranks than those of the incorrect and false positives. For examples, the re-ranking algorithm ZRANK [17] aims for a more accurate and quick re-ranking of the rigid docking predictions from ZDOCK (i.e. ZDOCK2.3 [9] at the time). Unlike ZDOCK2.3, the scoring function of which consists of grid-based discrete functions derived from both the receptor and the ligand [9], the scoring function developed in ZRANK includes a linear sum of the potential terms (e.g. van der Waals, electrostatics and desolvation) and does not require the grid-based presentations before the scoring [17]. ZRANK therefore does not significantly affect the overall complexity of the initial docking algorithm, marking it an important improvement in terms of computational efficiency because it can compute quickly and effectively the scores of each of the predicted protein complexes while there is no Fast Fourier Transform (FFT) technique required. In particular, ZRANK achieves noticeable improvement by improving the rankings of the top 2000 conformations for most of the cases in the protein complex benchmark 2.0 [16,17] and benchmark 4.0 over the initial docking programs ZDOCK (version 2.1, 2.3, and 3) (data not shown).

Another re-ranking technique using an implicit solvent model (Generalized Born or GB) implemented in a rigid docking program F^2^Dock also shows better performances by locating more correct predictions in higher ranks [18] than the conventional initial rigid docking. F^2^Dock applies a GB model to estimate the changes in the solvation energy (ΔEsol) at the protein surfaces of protein complexes, and uses this ΔEsol value to re-rank the docking predictions [18]. The F^2^Dock GB re-rank showed better results for two out of 3 types of protein complexes (i.e. Antigen/Antibody and Enzyme/Inhibitor) than those of the initial rigid docking ZDOCK3.0.2 [18]. The estimation of the ΔEsol in the GB model is dependent on the polarization energy, which represents the electrostatics changes due to the solvent. However this estimation neglects the conformational changes upon the complex formation [19]. Therefore biases may be expected due to the rigidity of the protein backbones and side-chains.

Although each of the techniques approaches from different angles of employing potential energy terms, they both interpret the solvent effect using the energy-based scoring functions and show significant improvement as compared to the initial docking algorithm ZDOCK3.0.2. It is observed that the inclusion of water effect into the protein interactions is taken into account [17,18]. However, the water is yet presented *implicitly*. This is not always true for all the protein complexes, especially for Antigen/Antibody complexes.

Water molecules are often found in protein-protein interfaces. Despite quick exchanges between interfacial water and the bulk solvent, some water-mediated interactions contribute directly to the stability of protein formation and specificity of protein recognitions [20]. For examples, Antigen/Antibody interfaces are prone to less hydrophobic while the Enzyme/Inhibitor interfaces are more hydrophobic. However, studies have shown that both types of complexes contain either wet or dry interfaces [20] and that wet interfaces with water-filled cavities maintain the close pack of the structures and facilitate the protein interactions via water-mediated hydrogen bonds or indirect interactions [21-24]. Although water plays an important role in protein interactions, inclusion of the water in current protein docking and re-ranking algorithms is only implicit due to the limited computational resources. In other words, the water molecules are not actually presented in the protein-protein interactions in those computational models.

Hence in this work, combining two objectives of simultaneously improving the ranks of the correct predictions and *explicitly *presenting the water influence on the protein interactions, we proposed a new re-ranking algorithm via an energy-based scoring function, namely IFACEwat. The IFACEwat was implemented based on the Interface Atomic Contact Energy (IFACE) of the initial rigid docking algorithm ZDOCK3.0.2 [25] and on derived energies of water-involved interactions at the protein interfaces. It was expected that when accounting exclusively for the water-mediated interactions and taking advantages of shape complementarity, we could elicit some extent of protein flexibility at the protein interfaces, especially for the Antigen/Antibody complexes, which could not or be limitedly achieved by some current initial rigid docking algorithms.

## Results and discussion

The proposed re-ranking algorithm is a combination of various potentials and energy terms, especially employing the interface Atomic Contact Energy (IFACE) of the initial docking algorithm ZDOCK3.0.2 [25] to evaluate both the protein recognitions and water mediated effect at the protein interfaces (see Methods). The algorithm, namely IFACEwat, was used to estimate the number of near native structures (or hits) and re-rank those hits, which were among the predicted complexes generated by the initial rigid docking algorithm ZDOCK3.0.2 (hereafter referred to ZDOCK for simplification or otherwise noted). The re-ranking process was performed for each complex in the dataset of 159 cases of Antigen/Antibody, Enzyme/Inhibitor, and other types, and then followed by comparisons with the ranking results of the initial rigid docking algorithm ZDOCK and those of two other current re-ranking techniques ZRANK [17] and F^2^Dock [18].

### The IFACEwat improves significantly the rank of the near native structures as compared to the initial rigid docking, especially for Antigen/Antibody complexes

**Figure 1:**
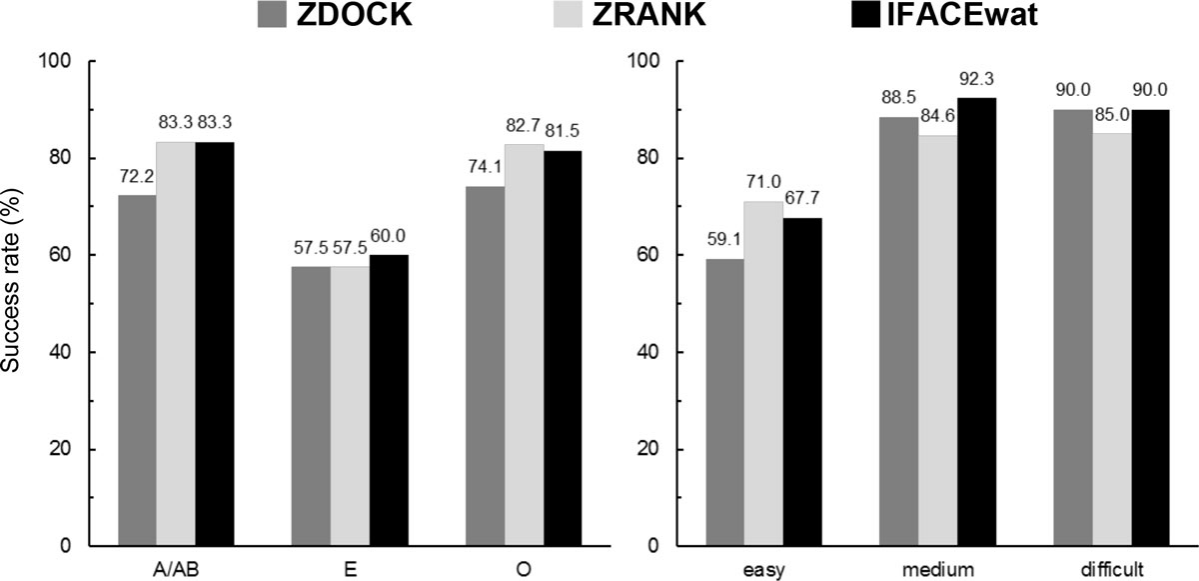
**Performances of ZDOCK, ZRANK, and IFACEwat re-ranking technique in terms of success rate of different complex types**. For example, on the left chart, the ZDOCK success rate of 72.2%(13/18) for the Antigen/Antibody complex (A/AB) means that in the top 100 predictions generated by ZDOCK, at least one hit was found in 13 out of 18 cases of type A/AB.

A dataset extracted from the latest protein-protein benchmark 4.0 [26] is used in this work. It includes non-redundant structures of protein-protein complexes of Enzyme/Inhibitor (E), Antibody/Antigen (A/AB), and other types (O) (see details in Methods section). Results of the initial dockings by ZDOCK showed that 139 out of 159 cases of this dataset, including 18 Antigen/Antibody, 40 Enzyme/Inhibitor, and 81 other complexes, obtained at least one near native structure among 2000 predictions. In our results, a near native structure called a "hit" was defined as the complex structure, the ligand orientation of which obtained a root mean square deviation (RMS) less than 2.5Å. The value of RMS ≤ 2.5Å is commonly used to indicate how deviating the predicted ligand orientation is as compared to the original ligand orientation in the corresponding crystalized structure. For this comparison, we used "success rate" to evaluate the performances (Figure 1).

The *success rate *reflects how well the technique can rank the correct predictions in the top ranks, and therefore it shows how effective the technique can discriminate the correct structures from the incorrect and false positive ones. It is important to achieve the higher ranks for the correct predictions because those top ranked predictions would then undergo further refinement using more intensive computing, e.g. molecular dynamics simulation.

$$\text{Success rate}=\frac{\#\text{ cases that obtain at least 1 hit in the top 100 predictions}}{\#\text{ total cases of the particular complex type}}$$

Comparisons of the success rate between ZDOCK and the IFACEwat indicated that the IFACEwat improved the ranks for most of the complexes classified either by physicochemical properties (Antigen and unbound/bound Antibody complex A/AB, Enzyme/Inhibitor E, and others O) or by CAPRI-defined difficulty level (i.e. easy, medium, and difficult) [26]. Except for the difficult cases, in which both the methods achieved a tight percentage of 90% (Figure 1 - right panel), the IFACEwat showed better ranking outcomes, and was therefore expected to discriminate better the correct from the false positive predictions.

In addition, it was demonstrated in Figure 1 (left panel) that the IFACEwat outperformed ZDOCK for A/AB complexes (83.3% as compared to 72.2% of the latter). The improvement was due to including interfacial water effect and the flexibility of the interfaces, which are the two important characteristics of the Antigen/Antibody interactions (i.e. most of Antigen/Antibody complexes contain wet-interfaces and conformational changes are induced during their complex formation [13,20]). For examples, in the two Antigen/Antibody cases of [PDB:1IQD] (easy) and [PDB:1BGX] (medium) the IFACEwat improved significantly both the number of hits and ranks of the hits (see Additional file 1). For both these two cases, while there was only one hit that could be generated, the IFACEwat could detect it and ranked it in the top 1, but ZDOCK could find none. Furthermore, for several difficult complex cases, while both the methods could find the same numbers of hits, the IFACEwat ranked the first hit in the better ranks (e.g. [PDB:2O2B] obtained 1 hit which was found in rank 7 by ZDOCK, but in rank 5 by the IFACEwat). Noticeably, the IFACEwat found 2 hits more for the difficult case of [PDB:1BKD] (Additional file 1).

**Figure 2:**
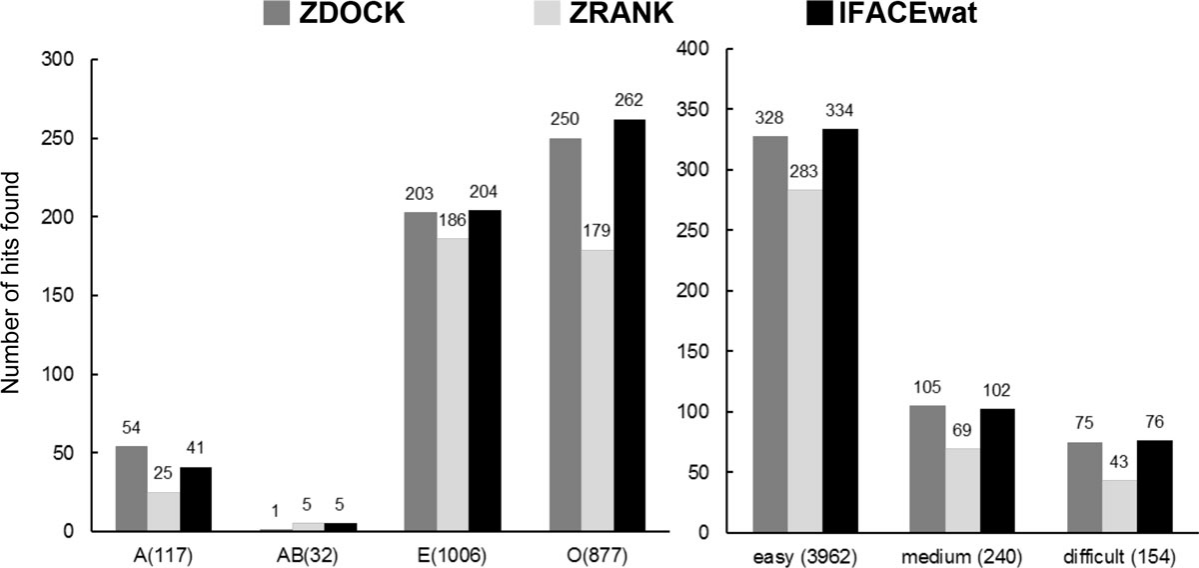
**Performances of ZDOCK, ZRANK, and IFACEwat in terms of numbers of hits found for each type of complexes**. The total number of hits found is shown in the brackets.

It was also observed that the IFACEwat could detect more near native structures than ZDOCK (Figure 2). Especially for the type AB (complexes of Antigen and the bound-Antibody), while ZDOCK was able to locate only one hit of 1 complex (i.e. [PDB:1K4C] shown in Additional file 1), the IFACEwat found 5 hits for 3 cases ([PDB:1K4C], [PDB:1IQD], and [PDB:1KXQ]) out of 7 cases and ranked the first hit as rank 1 for all these 3 cases.

Compared to ZRANK, the IFACEwat performed equivalently well for both the Antigen/Antibody (at the equal success rate of 83.3%) and Enzyme/Inhibitor complexes (60% versus 57.5% by ZRANK), yet slightly better for some medium and difficult cases (Figure 1 - right panel).

**Figure 3:**
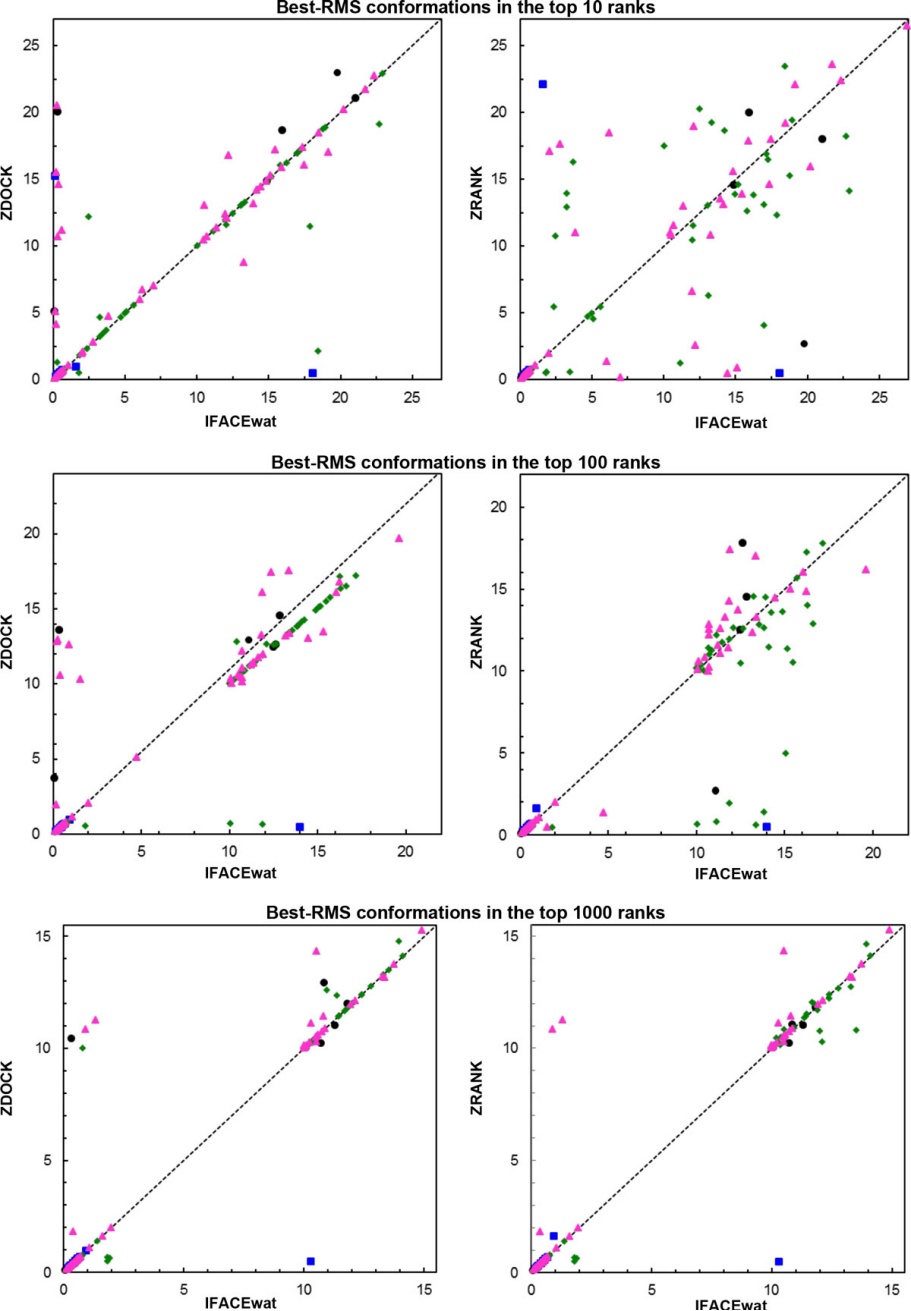
**Best RMS conformations in the top 10, 100, and 1000 ranks found by the IFACEwat against ZDOCK (left) and ZRANK (right)**. Each dot represents a protein complex of corresponding complex types: Antigen/Antibody (A in blue, AB in black), Enzyme/Inhibitor (E in green), Others (O in magenta).

To demonstrate further the performance comparisons among the three methods, we used the complex conformations that obtained the best RMS for the top 10, 100, and 1000 predictions (Figure 3 andAdditional file 2). It was indicated that, for either types of complex classification (easy, medium, difficult or Antigen/Antibody, Enzyme/Inhibitor, Others), the IFACEwat outperformed ZDOCK in locating the best RMS conformations (left panels in both the Figures). For the top 10 ranks, the two methods were equivalent although the IFACEwat had shown better performance particularly for complex type AB (Figure 3left). When the number of predictions increased to 1000, the trend was clearer for all the types, especially for medium and difficult cases (Additional file 2-left).

As comparing with ZRANK, the trend was also more pronounced when the number of predictions increased, especially for the medium and difficult cases (Additional file 2-right). However, both the methods performed equivalently well in locating the best RMS conformations for Antigen/Antibody cases, yet sometimes ZRANK was found with slightly better performance (Figure 3-right).

### Comparisons of the IFACEwat and F^2^Dock

While the IFACEwat was being developed, there was another re-ranking method independently developed and published in March 2013 and included in the F^2^Dock package [18]. The F^2^Dock applies Generalized Born model to estimate the changes in solvation energy of the protein interfaces, and also considers separately the characteristics of protein complexes.

**Figure 4:**
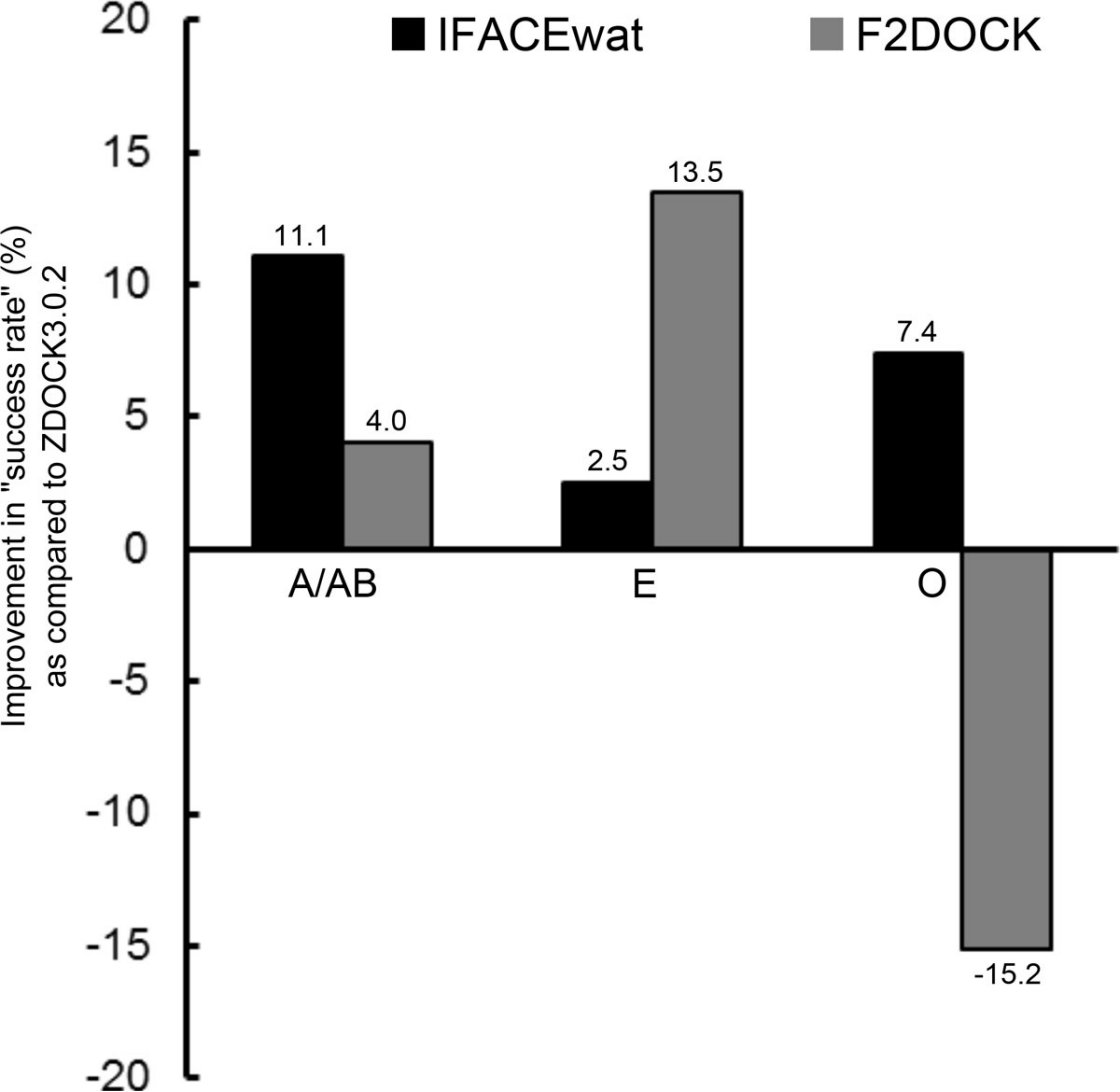
**Improvement of the IFACEwat and F2Dock against the initial docking ZDOCK**. The *success rate *is defined the same as in Figure 1. It is observed that the IFACEwat performs better for Antigen/Antibody (A/AB) complexes while the F^2^Dock is better for Enzyme/Inhibitor (E).

There are different implementations between the IFACEwat and F^2^Dock in ways of defining the near-native structures and re-ranking them [18]. For examples, while F^2^Dock defines the "hit" based on RMS of the interface-involved residues (with interface RMS ≤ 5Å), the "hit" in the IFACEwat is defined with the RMS of the whole ligand orientation against ligand position in the crystalized structures (with RMS ≤ 2.5Å). Therefore, to obtain a fair comparison, we evaluated the relatively significant improvement of the success rate (as defined above) achieved by each of the methods as compared to the initial docking program ZDOCK (Figure 4).

It was observed in Figure 4 that different implementations of the role of water in the scoring function might result in different performances between the two techniques. In particular, the IFACEwat performed better for some Antigen/Antibody cases (e.g. improved 11.1% over ZDOCK versus 4% by F^2^Dock) and also for some medium and difficult cases (see Additional File 3). Both the methods account for the water effect, but while the F^2^Dock approximates the changes of solvation energy only [18], the IFACEwat includes the free energy change of the complex interfaces by applying solvated rotamers, subsequently accounting for the side chain and backbone flexibility of the proteins; especially for some of difficult cases that required induced conformational fit during the protein associations.

The F^2^Dock on the other hand performed better than the IFACEwat in some Enzyme/Inhibitor cases (13.5% versus 2.5% in Figure 4). This might be the result of considering the energy to form the cavities that were frequently observed in the Enzyme and Inhibitor interactions.

Unlike F^2^Dock, a strict constraint of hit numbers is set in the IFACEwat implementation to ensure that the first hit needed to acquire the rank as high as possible. In fact, the IFACEwat estimates the numbers of hits that are considered only among the numbers of near-native structures in the ground truth. For example, for the case of [PDB:1BGX] (Antigen/Antibody, medium), there was only 1 hit generated according to the ground truth (so *number_of_hits = 1*). The constraint was set to the case that only the conformation ranked at the top-1 (and with RMS ≤ 2.5Å) would be considered as a hit by the IFACEwat. And this hit was the first and the only hit according to the method. Other conformations out of this window length of ranking (e.g. here *window_length = 1*) would not be considered as hits found by the IFACEwat although their conformations obtained RMS ≤ 2.5Å. On the other hand, F^2^Dock considered any conformations with interface-RMS ≤ 5Å as hits found [18].

As a result, some first hits found by F^2^Dock obtained low ranks, e.g. for the case of [PDB:1E6J] (Antigen/Antibody - type easy), the first hit was ranked at rank 126 by F^2^Dock, but ranked at rank 2 by the IFACEwat. However, for the [PDB:1BGX] case above F^2^Dock did not obtain any hit (see Additional file 3).

### The IFACEwat presented the water-mediated hydrogen bond network in the complex interfaces

In this work, the solvent effect is explicitly presented in the complex interfaces during the re-ranking. Complement with the interface Atomic Contact Energy (IFACE) of the initial docking ZDOCK, the water contribution via the energies of protein-water dispersion and water-mediated Hydrogen bonds fulfills the overall picture of water-facilitating interactions between the protein partners, particularly in the Antigen/Antibody complexes.

**Figure 5:**
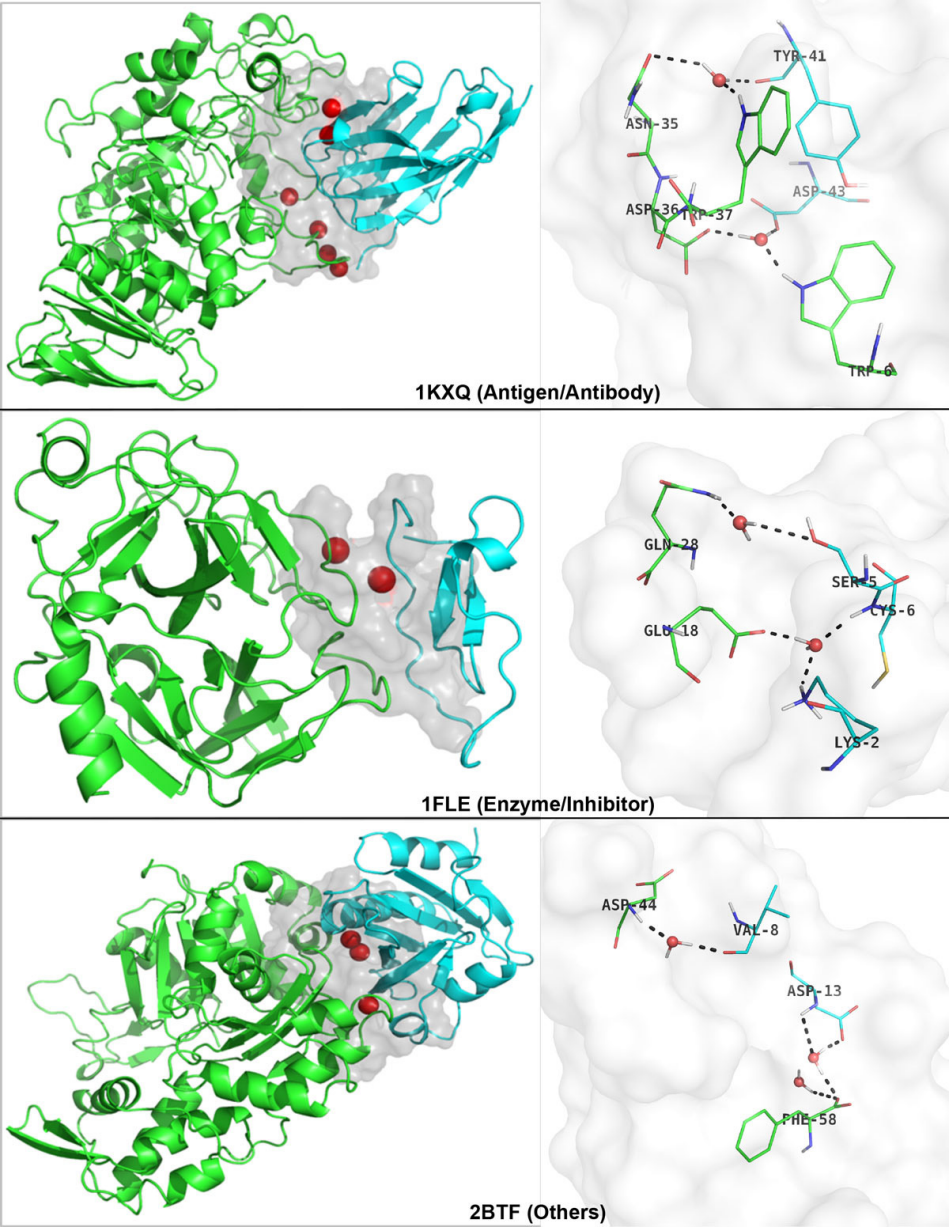
**Presence of the water-mediated Hydrogen bonds at the interfaces of protein complexes, the first hit of which is improved in ranking**. The complex interfaces (in grey shaded surfaces) contain interfacial water molecules (red spheres) that mediate the hydrogen bonds (black dotted lines) bridging the interactions between the receptor (green) and the ligand (cyan).

Especially, the IFACEwat is strictly constrained in defining the *hit *to locate the near native structures of protein complexes in as high ranks as possible (as describe above). Together with the inclusion of the interfacial water, the IFACEwat improves not only the number of hits found but also the ranks of the first hit, which represents the best complex predicted and the nearest to the corresponding original protein complex (as shown in Additional file 1). Among the protein cases that were improved in ranking the first hit as compared to the initial docking ZDOCK, structural observations of their complex interfaces revealed the presence of the hydrogen bond network, which was mediated by the interfacial water (Figure 5). For the Antigen/Antibody case of [PDB:1KXQ], while the initial docking could not locate any hit, the IFACEwat found the best hit (with RMS = 0.09Å) and ranked it as the top-1. The interface of this protein complex contained 6 water molecules, two of which contributed to the hydrogen bond network that bridged the protein interactions between the interfacial residues. Similarly, in the cases of [PDB:1FLE] (Enzyme/Inhibitor) and [PDB:2BTF] (Others), it was observed that the interfacial water molecules also formed the mediated hydrogen bonds that connected the protein chains (Figure 5).

### The IFACEwat improved the rank of the first hit for either with- or without water-containing structures

Our results of the re-ranking show that the IFACEwat also improves the number of near native structures and their ranks for several protein complexes that do not contain interfacial water (Table 1). In fact, the re-ranking effectiveness of the IFACEwat maintains for such cases because it takes into account the free energy change of the protein complex interfaces (i.e. ΔG_interface _in Equation 1 - in Methods section).

**Table 1:** The IFACEwat improves both the number of hits and the rank of the first hit for either protein complex cases of with- or without interfacial water.

Crystal structure contains WAT	Complex	Type	↑ number of hits		↑ rank of the first hit		
			
			ZDOCK	IFACEwat	ZDOCK	IFACEwat	∆G_interface_(1^st^hit)
No	1FC2	O	16	20	-	-	-
	4CPA	E	-	-	51	4	-26.14
	1QA9	O	-	-	2	1	-76.04
	2AYO	O	-	-	32	10	-47.54
	2HLE	O	-	-	2	1	-178.17
	2VDB	O	-	-	57	18	-58.42
	3BP8	O	_-_	_-_	11	3	-32.08

Yes	1DQJ	A	6	7	-	-	-
	1BGX	A	0	1	-	1	-348.73
	1K4C	AB	1	3	-	-	-
	1KXQ	AB	0	1	-	1	-191.97
	1CLV	E	140	141	-	-	-
	2O3B	E	-	-	7	5	-47.95
	1YVB	E	0	1	-	2	-55.84
	1FLE	E	-	-	6	4	-47.85
	1AK4	O	0	1	-	3	-44.51
	1FQJ	O	2	4	-	-	-
	1H9D	O	0	1	-	1	-131.85
	1HCF	O	0	1	-	1	-175.53
	1RV6	O	0	1	-	3	-110.72
	1T6B	O	3	5	-	-	-
	1Z0K	O	0	1	-	1	-133.32
	1Z5Y	O	2	3	-	-	-
	2A9K	O	0	1	-	2	-161.63
	2B4J	O	6	8	-	-	-
	1MQ8	O	4	5	-	-	-
	1R6Q	O	3	4	-	-	-
	2CFH	O	9	10	-	-	-
	2OZA	O	1	2	-	-	-
	2ZOE	O	6	8	-	-	-
	2BTF	O	-	-	6	1	-142.31
	2OOR	O	0	3	-	1	-141.03

"-" represents "*not applicable*" for the corresponding cases. For examples, in the first case [PDB:1FC2], it is found that IFACEwat improves the number of hits (i.e. 4 hits more than ZDOCK), but does not improve the rank of the first hit (e.g. the first hit found by both IFACEwat and ZDOCK is ranked at top-1). Also noted in this table that ∆G_interface _value is only shown for the first hit of the case, in which the rank of the first hit is improved by the IFACEwat.

In addition to highlighting the contributions of water in the interfaces, the IFACEwat accounts for the interactions occurring at the complex interfaces by including the free energy change as the last potential term (Equation 1). Re-ranking observations of some complex structures that do not contain water indicated that the contribution of the free energy change ΔG_interface _helps improve significantly the rank of the first hit.

Among the components that were integrated in this ΔG_interface _term, the energies of side chain- and backbone-involved hydrogen bonds contributed noticeable portions in the overall value of the ΔG_interface _and helped determine the influence of side chain flexibility in the interfaces. Except for the complex [PDB:4CPA] (Enzyme/Inhibitor) in Table 1 the interactions occurring at the interfaces of the other non-water protein complexes were induced by significant conformational changes [27-31]. Therefore, it was expected that taking into account these energy contributions would help distinguish the good-binding complexes from the others. For examples, in the two cases obtaining the first hit at top-1 rank, i.e. [PDB:1QA9] and [PDB:2HLE], the free energies ΔG_interface _were -76.04 kcal/mol and -178.17 kcal/mol, respectively. Their break-down of the hydrogen bond-involving energies of -2.805 kcal/mol and -5.712 kcal/mol, respectively, indicated the sufficient contributions of the hydrogen bonds in the protein interactions. However, those energies were ignored by the initial docking algorithm ZDOCK.

Similarly, for some water-containing complex cases, the first hit of which was ranked top-1 by the IFACEwat, the free energies of the complex interfaces also obtained significantly low values (as in the more negative the more favorable). Particularly, in 2 cases of Antigen/Antibody complexes, i.e. [PDB:1BGX] (ΔG_interface _= -348.73 kcal/mol) and [PDB:1KXQ] (ΔG_interface _= -191.97 kcal/mol), the IFACEwat located the first hit at rank 1 while ZDOCK could not find any hit. Therefore, it was suggested that including the consideration of the flexibility of protein side chains during the protein associations improved the discrimination of good binding complexes from the others, thereby improving the rank of the first hit or the best near native structure of the protein complexes.

### Free energy change (ΔG_interface_) plays the most important role in the IFACEwat scoring function

The IFACEwat scoring function includes various potential terms of the complex-binding score and the protein interactions at the interfaces (i.e. protein-protein and protein-water interactions). The contributions of these terms are evaluated given a set of weights (*w_1_, w_2_, w_3_, w_4_*), each of which reflects an independent influence of each potential onto the overall *f *score (Equation 1). As discussed more in detail in this section, the free energy change (ΔG_interface_) contributes the most among the four potential terms, especially in Antigen/Antibody complexes (i.e. *w_4 _= 6.2*). The free energy change (ΔG_interface_) is used to evaluate the interactions occurring at the complex interfaces, therefore determining the more favorable bound complex conformations (i.e. the more negative values).

To evaluate the contribution of each potential term, "leave-one-parameter-out" experiments were performed. In those experiments, each potential contribution *i *was alternately disabled, i.e. *w_i _= 0 *(Table 2), while the others were used to calculate the total *f *score and estimate the number of the found near native structures (or hits). Resulting observations show that the contribution of the free energy change (ΔG_interface_) plays the most important role among the potential terms. When *w_4 _= 0*, the protein-protein interactions that occurred at the interfaces were mostly ignored and the side chain flexibility was limited (in experiment #4), consequently leading to the noticeable drop of the total number of hits found (Table 2), especially for the Antigen/Antibody complexes (i.e. types A and AB obtained only 17.1% and 37.5% numbers of hits, respectively as compared to the experiment #0). Differences of Antibody conformations between the two types (unbound in A and bound in AB) might explain the decrease of the number of hits. For the Antigen/bound-Antibody structures (type AB), interactions of the Antibody and its possible substrate might have re-oriented the conformation of the Antibody itself, substantially leading to conformational changes before it interacts with the Antigen. The significant drop of the number of hits in type A implied that the free energy change ΔG_interface _provided significant influence to determine the number of near native structures for the Antigen/unbound-Antibody complexes.

**Table 2:** Decreases in the numbers of near native structures for all types of protein complexes

#	W_i _= 0	Number of hits found (in %)^a ^and level of hit decrease			
		
		A	AB	E	O
**0**	**(W_1_,W_2_,W_3_,W_4_)**	**100**	**100**	**100**	**100**
1	(0,W_2_,W_3_,W_4_)	↓↓↓↓ (41.5)	↓↓ (87.5)	↓↓ (73.8)	↓↓ (74.4)
2	(W_1_,0,W_3_,W_4_)	-	↓↓ (75.0)	↓ (97.1)	↓ (98.2)
3	(W_1_,W_2_,0,W_4_)	-	-	↓ (97.0)	↓ (98.0)
4	(W_1_,W_2_,W_3_,0)	↓↓↓↓ (17.1)	↓↓↓ (37.5)	↓↓ (78.0)	↓↓ (90.0)

5	(W_1_,0,0,W_4_)^b^	-	↓↓ (87.5)	↓ (97.0)	↓ (97.7)
6	(0,W_2_,W_3_,0)	↓↓↓↓↓ (4.87)	↓↓↓ (50.0)	↓↓↓↓ (30.0)	↓↓↓↓ (18.7)
7	(0,0,0,W_4_)	↓↓↓↓ (43.9)	↓↓ (75.0)	↓↓ (71.9)	↓↓ (75.8)
8	(W_1_,0,0,0)	↓↓↓↓ (17.0)	↓↓↓↓↓ (0.0)	↓↓ (74.2)	↓↓ (89.0)

Antigen/unbound-Antibody (A), Antigen/bound-Antibody (AB), Enzyme/Inhibitor (E) and others (O): ↓ (less than 5% hits drop), ↓↓ (less than 30% hits drop), ↓↓↓ (noticeably drop less than 50% hits), ↓↓↓↓ (significantly drop less than 70% hits), ↓↓↓↓↓ (dramatically drop more than 95% hits), - (unchanged). Experiment #0 shows the results of number of hits obtained from the full set of weights (*w_1_, w_2_, w_3_, w_4_*) for each complex type and is used as the reference (in bold). ^a^% of hits as compared to the reference (i.e. results of the experiment #0) ^b^Only 4 combinations (#5 to #8) of weights are presented to highlight the significant contributions of w_1 _and w_4_

Further, results of both the experiments (#4 and #6) with *w_4 _= 0 *showed the significant decrease of the number of hits of the types A and AB. Results of the Enzyme/Inhibitor complexes also indicated the similar decreasing trend, especially when both the first potential term (e.g. shape complementarity) and the free energy change (ΔG_interface_) were disabled in experiment #6, the results of which significantly became worse. In the experiment #6, numbers of hits obtained for each complex type were 4.87% (A), ~50% (AB), ~30% (E), and 18.7% (O) as compared to the results of experiment #0. Similarly, results of the experiment #8 reinforced the important role of the free energy change (ΔG_interface_) in the IFACEwat scoring function, particularly for the Antigen/Antibody complexes. Decrease of the number of hits therefore implies the increases of the false positives when the unfavorable complex conformations are not detected.

It was observed in Table 2 that restriction of the combinations of shape complementarity, electrostatics and desolvation also contributed significantly to the decrease of the number of hits for the type A complexes. When *w_1 _= 0 *in the experiment #1, except for type A (41.5%), most of the complexes obtained only around >70% (nearly 30% drops) of the number of near native structures as compared to the experiment #0 results. Comparisons of the two experiments #1 (with *w_1 _= 0*) and #4 (with *w_4 _= 0*) again revealed the different influences of both shape complementarity and free energy change on protein complexes of different physicochemical properties. Although the shape complementarity surprisingly affected much on the complexes type A (i.e. more than 50% drop) as shown in experiment #1, ignoring the contribution of the free energy change (ΔG_interface_) resulted in more decrease of hits (i.e. more than 82% drop shown in experiment #4). A similar decreasing trend was observed in type AB in experiments #1 and #4 as well as in the experiments #7 and #8. On the other hand, the shape complementarity showed more predominant role in determining the number of hits in the Enzyme/Inhibitor (type E) complexes.

It was also observed in the Antigen/Antibody complexes that the effect of water molecules was clearer on the complexes of type AB than on the complexes of type A. As discussed above, interactions of the Antigen with the bound-conformation of the Antibody might result in more spacious interfaces where the space could be occupied by the water molecules, thereby increasing the effect of water onto the protein interactions at the complex interfaces of type AB. Hence, when *w_2 _= 0 *(experiment #2), especially if there existed water molecules at the complex interfaces, this might cause the decreases in the number of hits in type AB, but not in type A.

### Optimal weights w_1_, w_2_, w_3_, w_4 _of the proposed scoring function

We performed various experiments to investigate the dependency of different potential terms on physicochemical properties of the protein complexes (data not shown). It was indicated that interactions between the Enzymes and the Inhibitors were driven mostly by hydrophilic and planar residues and that methods based on the Atomic Contact Energy did not likely work well in predicting interactions of Antigen/Antibody complexes. Because the dataset used in this work contained different types of protein complexes, i.e. Antigen/Antibody (A), Antigen/bound-Antibody (AB), Enzyme/Inhibitor (E), and Others (O), it was expected that contributions of the four potential terms in the Equation 1 (see Methods) would be diverse. Therefore, we estimated the weights *w_1_, w_2_, w_3_*, and *w_4 _*of the proposed scoring function (Equation 1) differently according to the different complex types.

After a number of iterations (averagely 40,000 steps) to estimate the weights using Monte Carlo method, the sets of weights [w1, w2, w3, w4] obtained for each complex type are [0.96, 0.002, 0.6, -6.2] for type A, [0.96, 0.95, 0.6, -6.2] for type AB, [1.6, 0.12, 0.15, -1.6] for type E and [2.0, 0.12, 0.15, -1.6] for type O.

Among the four weights evaluating the interactions of the protein complexes, *w4 *is set negative to maintain the positive *f *scores (Equation 1). The free energy change ΔG_interface _represents the interactions of the protein partners at the interfaces. It is considered as a complementary potential terms to either (1) the Score_IFACE_, which favors the shape complementarity of the two molecules, or (2) the complex flexibility due to the trade-off of the rigid docking. The more negative ΔG_interface _will favor the more stable interactions (ΔG < 0) and also reflects the difficulty level that drives the two molecules to interact with each other, e.g. *w_4 _= -6.2 *for type A/AB as compared to *w_4 _= -1.6 *for type E/O.

In fact, different sets of weights implicitly reflect different physicochemical properties between the Antigen/Antibody and Enzyme/Inhibitor complexes. In addition to the noticeable difference of *w_4 _*between the two types, the weight of the first term *w_1 _*also indicates the susceptibility to shape complementarity of type E/O (*w_1 _= 1.6 or 2.0*) over the other type A/AB (*w_1 _= 0.96*). The Enzyme-Inhibitor interactions are characterized as geometrical matching or shape complement whereas the Antigen and Antibody interactions are known as "induced fit" and require conformational changes during the associations. These properties however could not be totally interpreted using rigid docking although some "soft" docking techniques were applied (i.e. adjusted surface layers for receptor and ligand when their grids were prepared; some allowed slight overlaps, or the added electrostatics terms in the scoring function, etc. [9,10,32]).

While there are no significant differences of weight values between type E and O (in both of which the shape complementarity was more predominant), noticeable difference of weights is observed between type A and AB cases of Antigen/Antibody complexes. Especially, the Lennard-Jones repulsive energy contributes more dominantly in type AB (*w_2_=0.95*) than in type A (*w_2_=0.002*). Structural observations reveal that steric constraints of the Antibody conformations between the unbound (A) and bound (AB) might lead to the difference. Interactions of the Antigen with the Antibody bound-conformation (AB) might have formed more spacious interfaces where there is room for the presence of water. In fact for the AB structures, interactions of the Antibody and its substrate might re-orient the overall conformational packing and therefore expose more hydrophilic regions on its surface; consequently forming hydrophilic cavities when interacting with the Antigen. Increase of the hydrophilicity in those cavities might facilitate occupation of one or more water molecules, substantially increasing the water effect onto the interfacial free energy change of these AB complexes.

### The IFACEwat maintained sufficiently the computational efficiency yet improved ranking of the first hit

The IFACEwat scoring function was developed as a re-ranking technique using explicit interfacial water to further discriminate the correct predictions from the other incorrect and false positive ones of an initial docking algorithm, particularly in improving the results of predicting complex formations for Antibody/Antigen complexes.

The nature of the rigid initial docking is to keep the receptor rigid and allows limited flexibility of the ligand in order to achieve computational efficiency due to the large sizes of both the molecules. However, at the same time, it limits the ability of finding the correct bindings of the protein complexes as well as increases the probability of generating the false positive ones. This leads to a need of structural refinement to optimize the conformations of the protein complexes and further eliminate the decoys. Nonetheless, such a process, e.g. using energy-based evaluation by MD simulation, for the refinement purposes requires intensive computation, especially for the Antibody/Antigen complexes. Therefore, for this work the IFACEwat is expected to reduce as many as possible false positives that need not to be refined before heading to the refinement steps, but still maintain the computational efficiency.

Results of re-ranking time (*T_R_*) by the IFACEwat (Table 3) indicate that the IFACEwat is able to maintain sufficient timing (less than approximately 22 minutes) to re-rank 2000 conformations of each protein complex. Among the complexes, two Antigen/Antibody complexes, i.e. [PDB:1BGX] and [PDB:1KXQ] took the longest re-ranking time using the IFACEwat. While the IFACEwat ranked their first hits as the top-1, the initial docking ZDOCK could not locate any hits.

**Table 3:** Real running time *T_R _*of the IFACEwat in re-ranking some of the protein complexes and the estimated formula of the MD time for the structural refinement stage of the IFACEwat results as compared to ZDOCK

Complex	Type	IFACEwat re-ranking time *T_R _*(second/~minute)	Expected MD refinement time^b ^to obtain at least 1 near native structure using			
			
			ZDOCK		IFACEwat	
			
			Rank of 1st Hit	MD time	Rank of 1st Hit	MD tìme
4CPA	E	489.9/8.2	51	51 * T_Di_	4	4 * T_Di_
1QA9	O	542.9/9.1	2	2 * T_Di_	1	T_Di_
2AYO	O	1109.8/18.5	32	32 * T_Di_	10	10 * T_Di_
2HLE	O	819.0/13.6	2	2 * T_Di_	1	T_Di_
2VDB	O	864.0/14.4	57	57 * T_Di_	18	18 * T_Di_
3BP8	O	500.8/8.3	11	11 * T_Di_	3	3 * T_Di_

1BGX	A	1215.4/20.2	-	2000 * T_Di_	1	T_Di_
1KXQ	AB	1289.5/21.5	-	2000 * T_Di_	1	T_Di_
2O3B	E	632.4/10.5	7	7 * T_Di_	5	5 * T_Di_
1YVB	E	855.5/14.2	-	2000 * T_Di_	2	2 * T_Di_
1FLE	E	564.0/9.4	6	6 * T_Di_	4	4 * T_Di_
1AK4	O	290.4/4.8	-	2000 * T_Di_	3	3 * T_Di_
1H9D	O	954.9/15.9	-	2000 * T_Di_	1	T_Di_
1HCF	O	738.7/12.3	-	2000 * T_Di_	1	T_Di_
1RV6	O	848.2/14.1	-	2000 * T_Di_	3	3 * T_Di_
1Z0K	O	859.6/14.3	-	2000 * T_Di_	1	T_Di_
2A9K	O	678.9/11.3	-	2000 * T_Di_	2	2 * T_Di_
2BTF	O	1130.1/18.8	6	6 * T_Di_	1	T_Di_
2OOR	O	1297.8/21.6	-	2000 * T_Di_	1	T_Di_

^a^Re-ranking of 2000 predictions of each protein complex on an Intel Xeon CPU E5540@2.53GHz machine. ^b^Assume that each conformation of each complex takes equivalent time during the MD simulation, i.e. T_Di_. "-" means ZDOCK does not obtain any hit in the corresponding case

Let *T_Di _*be the MD simulation time for each conformation that would undergo the structural refinement and assume that each of them would take an equivalent time. In the cases of [PDB:1BGX] and [PDB:1KXQ], there would be 2000*T_Di _computational time needed for the MD simulation to find the near native complex structure. However, after being re-ranked by the IFACEwat, only 1*T_Di _simulation time would be needed to obtain at least one near native structure of the protein complex because this conformation was found at the top-1. Similarly for other complexes, which obtained the higher rank for the first hit, the speed of MD simulation would be significantly improved if the structures underwent the refinement stage (as shown in Table 3).

## Conclusions

The re-ranking algorithm IFACEwat improves the initial rigid docking results by increasing not only the number of near-native structures found but also the ranks of the correct predictions. In fact, most of the near-native structures are ranked at the top-1.

The IFACEwat achieved a success rate of 83.8% for Antigen/Antibody complexes, which was 10% better than ZDOCK3.0.2. As compared to another re-ranking technique ZRANK, the IFACEwat obtained success rates of 92.3% (8% better) and 90% (5% better) respectively for medium and difficult cases. When comparing with the current re-ranking method in F^2^Dock, which employs a Generalized Born (GB) model in its energy-based function, it was shown that the IFACEwat performed equivalently with or even better than the GB-rerank F^2^Dock, especially for the Antigen/Antibody complexes. Our results showed that the improvement of those cases was facilitated by taking into account some extent of protein side chain flexibility when the proteins interacted, especially for those of difficult cases that required induced conformational fit during the protein associations.

Although the IFACEwat achieved a high success rate of 83.3% for Antigen/Antibody complex, 60% for Enzyme/Inhibitor, and more than 90% for medium and difficult cases, the method was restricted to improve particularly the result of the rigid docking algorithm ZDOCK3.0.2 and especially for the Antigen/Antibody complexes. In the near future, we will be further developing the algorithm to apply for other shape complementary-based docking algorithms to achieve better robustness.

## Methods

### Data preprocessing

In this project, we extracted a dataset from the latest Zlab protein-protein benchmark 4.0 [26] and retrieved the protein structures from Protein Data Bank [33]. The dataset includes non-redundant structures of protein-protein complexes of Enzyme/Inhibitor (E), Antibody/Antigen (A/AB), and other types (O). These structures are also classified into 3 groups (easy, medium, and difficult) according to the CAPRI [34] defined difficulty level that the current protein-protein docking algorithms could handle [26].

As we were interested in the interfaces of one-receptor versus one-ligand complexes, we only selected the complexes that contained monomer ligands from the benchmark 4.0 and included them in the dataset, which yielded a total of 159 cases. The dataset contains 113 easy cases (for which simple rigid-body docking algorithms can successfully predict the protein complex structures), 26 medium, and 20 difficult cases (predictions of which were significantly deviated from the native structures, i.e. interfacial root mean square deviation iRMS > 2.5Å [26]).

Initially, we quantified the statistics of interfacial water contributed in each of the crystalized protein complexes to investigate the contribution of water on the protein interactions in the original structures. It was observed that a percentage of 67% (130/159 complexes) of the dataset included water. Among the difficult cases, 16 out of 20 cases contained at least 6 water molecules buried in the protein interfaces. Hence, it was expected that taking into account the interfacial water molecules and water-mediated interactions would contribute significantly to the prediction of the protein-protein associations.

### Implementation of interfacial water into the proposed energy-based scoring function of IFACEwat

**Figure 6:**
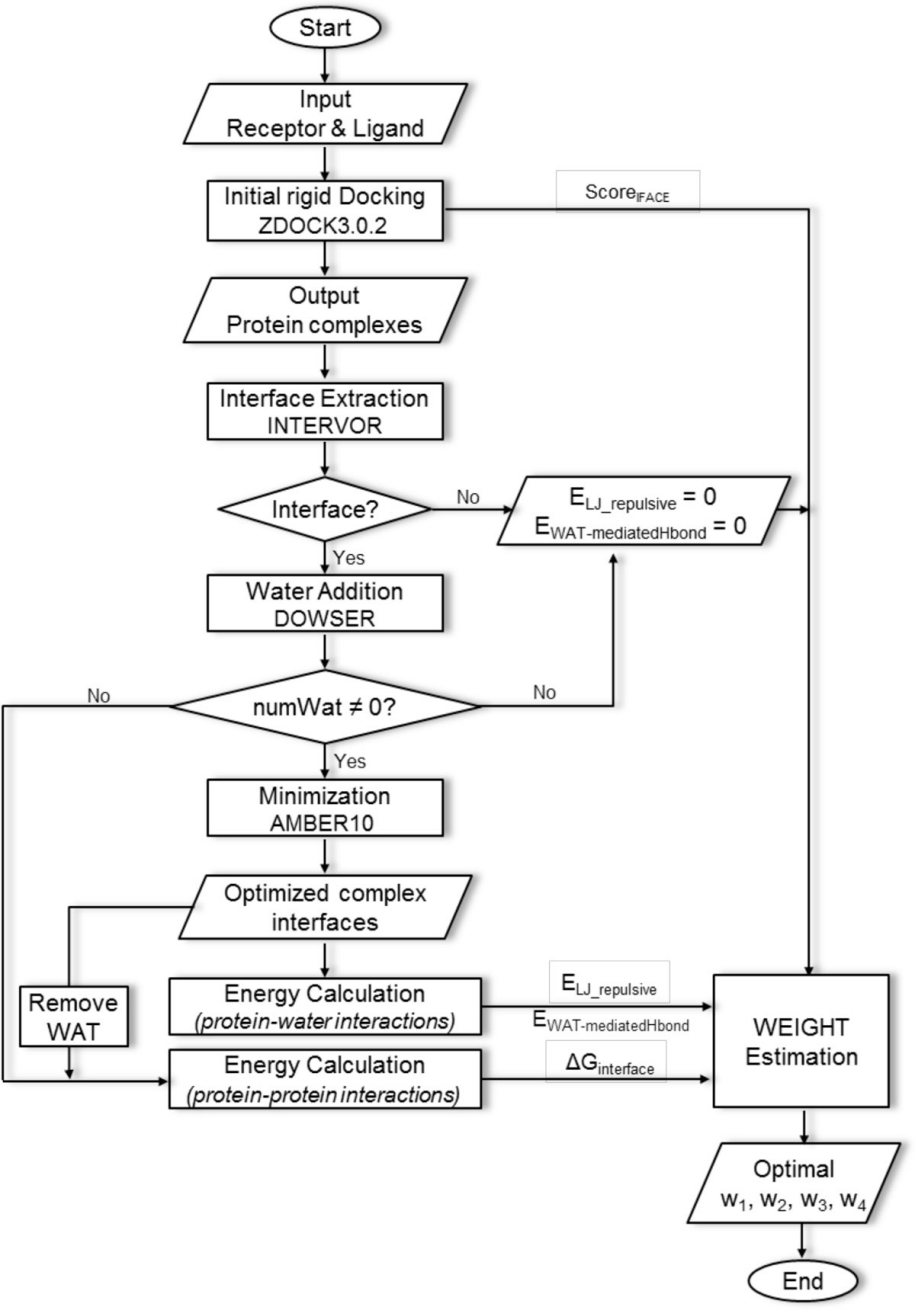
**Overview of the IFACEwat implementation**.

We proposed a new energy-based scoring function to re-rank the results of the initial rigid docking algorithm ZDOCK3.0.2. The scoring function contains a linear combination of potential scores and derived energies that are involved in the protein-protein and protein-water interactions at the complex interfaces (Equation 1).

(1) $$f=w_{1}\text{Scor}{\text{e}}_{\text{IFACE}}+w_{2}{\text{E}}_{\text{LJ\_repulsive}}+w_{3}{\text{E}}_{\text{water - mediated - Hbond}}+w_{4}\Delta {\text{G}}_{\text{interface}}$$

in which the Score_IFACE _is derived from the combination of shape complementarity, electrostatics, and desolvation (SC+ELEC+DE_IFACE _using the interface Atomic Contact Energy IFACE) employed in the initial rigid docking algorithm of ZDOCK3.0.2. The E_LJ_repulsive _and E_water-mediated-Hbond _are Lennard-Jones repulsive and water-mediated Hydrogen bond energies respectively to represent the protein-water interactions. Finally the ΔG_interface _is the free energy change of the interface representing the interactions between the protein partners. The four parameters w1, w2, w3, and w4 are separate weights of the corresponding potentials. Flowchart of the whole process is shown in Figure 6.

#### i Combinations of pairwise shape complementarity, electrostatics, and desolvation

ZDOCK3.0.2 is the rigid docking algorithm to optimize functions of shape complementarity (SC), electrostatics (ELEC), and desolvation (DE) as linear combination of correlations using FFT algorithm [35]. The ZDOCK3.0.2 algorithm employs the Interface Atomic Contact Energies (IFACE) for its desolvation potential [25,36] to represent the water effect during the protein associations. In this work, we estimated the sum of the three potentials (SC, ELEC, DE) and defined the score as Score_IFACE _= *w*_5_SC + *w*_6_ELEC + *w*_7_DE_IFACE_, in which *w_5 _= 0.01, w_6 _= 0.06*, and *w_7 _= 1 *[36]. For the purpose of improving the docking results of the rigid docking algorithm ZDOCK3.0.2, we initially kept these parameters intact.

The searching space was obtained from 15^o ^rotational sampling, which yielded 3,600 predictions. We selected top 2,000 predictions for the further implementation. In this work, we set the searching exhaustively to cover the entire binding regions of the ligand interfaces, which involved only the interacting residues with respect to the receptor. The purpose of doing this was to minimize the chances of getting false positives which were the wrong predictions but obtained high ranks by the ZDOCK3.0.2.

Observations of the FFT-based docking algorithms indicated that the Score_IFACE _was obtained based on correlations of two grid-based discrete functions of the ligand and the receptor. In order to simplify the calculation to achieve better computational efficiency, instead of following the FFT-based calculations, we chose to estimate the sum of all the energy terms linearly as shown in the Equation 1 and weight them separately. We therefore estimated firstly the Score_IFACE _before heading to other energy implementations.

#### ii Water-mediated contacts and the water effect on the protein interfaces

The water effect and contacts are represented by the energies of the protein-water dispersion (E_LJ_repulsive_) and the water-mediated Hydrogen bonds at the interfaces (E_water-mediated-Hbond_). Initially, we extracted the complex interfaces and subsequently followed a process of adding water molecules. There was no water molecule found in the interfaces of the predicted complexes by ZDOCK3.0.2 because no water was so far involved in the previous dockings of the protein complexes.

We extracted the complex interfaces using INTERVOR [37], a Voronoi-based algorithm modeling and computing macromolecular interfaces. Repeated interface extractions for all the crystalized complexes in the dataset were firstly performed and then mapped back to the original structures to confirm the reliability of the INTERVOR package. Our preliminary results showed that 159/159 interfaces of the protein complexes were successfully extracted and superimposed with the original crystalized structures. Only after being cross-confirmed of its fidelity, INTERVOR was used to extract the complex interfaces of all the 2000 predictions in the same protocol for each protein complex in the dataset.

Then, we applied DOWSER to explicitly add water molecules into the protein complex interfaces. DOWSER is a modeling package that investigates free energy of hydrophilic cavities and surfaces of proteins and adds water molecules into the cavities in which water exists [38]. Similarly, we tested the reliability and accuracy of the DOWSER application in recovering internal water of the protein interfaces by performing a simulation of adding water into interfacial cavities of protein complexes for the dataset of the 159 crystal structures. It was demonstrated that 91.3% of internal water molecules were successfully recovered (where the predicted water positions were within a distance ≤ 1.5Å from the water positions in the crystal structures). Therefore, it suggests that DOWSER could be a reliable tool to recover the water in the protein crystalized structures and thus it was used to predict the water positions for the interfaces of protein complexes in our work.

After adding water into each of the extracted interfaces of the protein complexes, we conducted the structural minimization using AMBER10 (force field *ff99SB*) to stabilize the water positions with respect to the whole structure of the protein interface. Initially, 2500 steps of steepest descent were performed and followed by another 500 steps of conjugate gradient minimization. During the minimization, no restraint was applied and random initial velocities were used to maintain the robustness. The minimized water-containing structures of the protein interfaces were then subject to the next process of energy calculations.

### Protein-water dispersion: E_LJ_repulsive_

In the presence of the interfacial water, there exist repulsive forces between the water molecules (oxygen atom) and any other atoms in a distance of 3.6Å (which is the maximum distance of the equilibrium inter-nuclear separation between oxygen and any atom). In this work, we presented contribution of the force to the energy changes of the protein interfaces by using the Lennard-Jones repulsive energy (E_LJ_repulsive_), and derived it from the linear van der Waals repulsive potential:

$${\text{E}}_{\text{LJ\_repulsive}}=4\varepsilon {\left(\frac{\sigma }{r}\right)}^{12}$$

where ε is the well-depth, r (with r ≤ 3.6Å) is the inter-nuclear distance, and σ is the sum of van der Waals radii of water (1.4Å) and the interacting atom (Table 4).

**Table 4:** van der Waals parameters (based on AMBER force field) used to calculate the Lennard-Jones repulsive energy in this project

Atom	σ(Å)	ε(kcal/mol)
C	1.7	0.173
N	1.55	0.179
O	1.52	0.2
S	1.8	0.2
H	1.2	0.063

### Water-mediated Hydrogen contacts: E_water-mediated-Hbond_

A solvated rotamer library developed by Jiang *et al*. offers this project a modeling approach to evaluate water impact on the protein interfaces and to describe water-mediated Hydrogen bonds [39]. Studies have shown that water attaching to protein side chains and mediating the Hydrogen bond network at protein interfaces contribute significantly to the changes of free energies in protein interactions [40,41], subsequently stabilizing structures of protein complexes. Therefore, it was expected that accounting for the interfacial water effect and energies of the water-mediated hydrogen bonds into the proposed scoring function would help locate effectively the favorable protein bindings, thereby improving the ranks of the correct predictions of protein complexes.

According to this library, a hydrogen bond that is considered as water-mediated needs to satisfy a number of geometric constraints of angles and distances around the water molecules (shown in Additional file 4). The water-mediated Hydrogen bond energy derived in this work is therefore distance-dependent and follows the energy function below:

$${\text{E}}_{\text{water - mediated - Hbond}}=\left\{\begin{array}{cc} {\text{E}}_{\text{Hb}} & \text{when constraints are satisfied}\\ 0 & \text{otherwise} \end{array}\right)$$

where E_Hb _is the Hydrogen bond energy evaluated by Jiang *et al*. from the solvated rotamer library.

### Free energy change of the protein interfaces ΔG_interface_

The last energy term in the proposed scoring function (Equation 1) represents interactions of protein partners at the interfaces. In order to take into account the flexibility of the interfaces and the trade-off of the rigid docking, we included all the interactions that involved side-chains. Derived from the solvated rotamers by Jiang *et al*. [39], the free energy change of the interface (ΔG_interface_) includes van der Waals interactions of protein atoms, hydrogen bond energies involved in side chain-backbone, side chain-side chain, backbone-backbone, and a potential of side-chain and backbone torsions. Further in this calculation, we included the solvation energy to complement the desolvation term (DE in the Score_IFACE_). The calculation of this ΔG_interface _term was performed using PyRosetta [42], the Rosetta package on the Python platform.

### Estimation of weights w_1_, w_2_, w_3_, w_4_

The proposed scoring function to re-rank the protein complexes (Equation 1) contains four separate weights *w_1_, w_2_, w_3_*, and *w_4 _*representing different contributions of the four potential terms. Optimal values of these weights were obtained by performing Monte Carlo experiments for the whole dataset of 159 protein complexes which were clustered into four types of complexes, i.e. Antigen/Antibody (A), Antigen/bound-Antibody (AB), Enzyme/Inhibitor (E), and Others (O). The weights were estimated during the recurring experiments until the maximal numbers of hits converged. In this project, hits were defined as the near native structures, RMS of which was within 2.5Å from the crystalized structures (RMS ≤ 2.5Å).

## List of abbreviations

IFACE: interface Atomic Contact Energy,

A/AB: Antigen/Antibody complex (A: unbound-Antibody, AB: bound-Antibody),

E: Enzyme/Inhibitor complex, O: other complex types,

RMS: root mean square deviation,

SC: shape complementarity, ELEC: electrostatics, DE: desolvation,

E_LJ_repulsive_: Lennard-Jones repulsive energy, E_water-mediated-Hbond_: water-mediated hydrogen bond energy,

ΔG_interface_: free energy change at the protein interface.

## Competing interests

The authors declare that they have no competing interests.

## Authors' contributions

CTTS and TDN carried out the experiments. CTTS, TDN, JZ, and CKK wrote the manuscript. All authors read and approved the final manuscript.

## Supplementary Material

Additional file 1 **Overall results of ZDOCK, ZRANK, and the IFACEwat in terms of numbers of near-native structures (hits) found and the rank of the first hit**. Cases which are in bold indicate that IFACEwat method performs equivalently with or better than both of the others. Click here for file

Additional file 2 **Best RMS of conformations in the top 10, 100, and 1000 ranks found by the IFACEwat against ZDOCK (left) and ZRANK (right)**. Each dot represents a protein complex case according to difficulty level: easy (black), medium (red), and difficult (blue). Click here for file

Additional file 3 **Re-rankings results that are equivalent with or better than the F^2^Dock-GB rerank**. To maintain a rational assessment, results between each of the methods and ZDOCK3.0.2 separately are used as the intermediate comparison since all the 3 methods are applied for the same dataset of protein complexes (the benchmark 4.0) with 15^o ^rotational sampling. Results of F^2^Dock are done by Chowdhury et al. Filename: AdditionalFile-3.pdf Click here for file

Additional file 4 **Angle and distance constraints to define a water-mediated Hydrogen bond according to the solvated rotamer library by Jiang *et al. ****d *is the distance between the water (W) oxygen and polar (acceptor A/donor D) atoms. H and AB are Hydrogen and Acceptor-Base atoms respectively. Click here for file

## Declarations

Publication of this article was funded by MOE AcRF Tier 2 Grant ARC39/13 (MOE2013-T2-1-079), Ministry of Education, Singapore.

This article has been published as part of *BMC Bioinformatics *Volume 15 Supplement 16, 2014: Thirteenth International Conference on Bioinformatics (InCoB2014): Bioinformatics. The full contents of the supplement are available online at http://www.biomedcentral.com/bmcbioinformatics/supplements/15/S16.
