# Supplementary material for: IFACEwat: the interfacial water-implemented re-ranking algorithm to improve the discrimination of near native structures for protein rigid docking
Source: BMC Bioinformatics. 2014 Dec 8;15(Suppl 16):S9. doi: 10.1186/1471-2105-15-S16-S9 (PMC4290663; doi:10.1186/1471-2105-15-S16-S9)
Supplement: Additional file 1 — Overall results of ZDOCK, ZRANK, and the IFACEwat in terms of numbers of near-native structures (hits) found and the rank of the first hit. Cases which are in bold indicate that IFACEwat method performs equivalently with or better than both of the others. [file 1471-2105-15-S16-S9-S1.pdf]

Additional file 1: Overall results of ZDOCK, ZRANK, and the IFACEwat in terms of numbers of near-native structures (hits) found and the rank of the first hit. Cases which are in bold indicate that IFACEwat method performs equivalently with or better than both of the others.

Complex	Type	ZDOCK		ZRANK		IFACEwat		
		#hits	rank of 1st hit	#hits	rank of 1st hit	#hits	rank of 1st hit	% (#hits/total hits)
Easy (113)								
1AHW	A	3	3	2	1	0	-	0
1BVK	A	5	1	0	-	1	2	10
**1DQJ**	**A**	**6**	**1**	**1**	**2**	**7**	**1**	**41.18**
1E6J	A	1	1	1	1	1	2	33.33
1JPS	A	2	1	2	1	1	1	25
1MLC	A	11	1	7	1	6	1	30
1VFB	A	2	1	1	4	2	4	25
**1WEJ**	**A**	**6**	**1**	**2**	**1**	**6**	**1**	**31.58**
**2FD6**	**A**	**1**	**1**	**1**	**1**	**1**	**1**	**25**
2I25	A	14	1	5	1	13	1	68.42
2VIS	A	2	1	1	2	1	3	33.33
1FSK	AB	0	-	0	-	0	-	-
**1IQD**	**AB**	**0**	**-**	**1**	**1**	**1**	**1**	**100**
**1K4C**	**AB**	**1**	**1**	**3**	**1**	**3**	**1**	**42.86**
**1KXQ**	**AB**	**0**	**-**	**1**	**1**	**1**	**1**	**16.67**
1NCA	AB	0	-	0	-	0	-	-
1NSN	AB	0	-	0	-	0	-	0
2JEL	AB	0	-	0	-	0	-	0
**1AVX**	**E**	**4**	**1**	**4**	**1**	**4**	**1**	**36.36**
1AY7	E	0	-	0	-	0	-	-
1BVN	E	10	1	9	1	9	1	28.13
1CGI	E	0	-	0	-	0	-	0
**1CLV**	**E**	**140**	**1**	**119**	**1**	**141**	**1**	**33.98**
1D6R	E	0	-	0	-	0	-	0
**1DFJ**	**E**	**3**	**1**	**2**	**1**	**3**	**1**	**27.27**
1E6E	E	1	1	3	1	1	1	11.11
1EAW	E	3	3	0	-	2	2	5.56
1EWY	E	0	-	0	-	0	-	0
1F34	E	0	-	0	-	0	-	-
**1FLE**	**E**	**1**	**6**	**0**	**-**	**1**	**4**	**8.33**

1GL1	E	0	-	0	-	0	-	0
**1GXD**	**E**	**3**	**1**	**3**	**1**	**3**	**1**	**50**
1HIA	E	0	-	0	-	0	-	0
1JTG	E	3	1	4	1	3	1	37.50
1MAH	E	0	-	1	1	0	-	0
1N8O	E	0	-	0	-	0	-	-
1OC0	E	0	-	1	29	0	-	0
1OPH	E	4	1	2	2	3	1	37.50
1OYV	E	0	-	0	-	0	-	-
1PPE	E	2	25	0	-	2	30	4
1R0R	E	1	38	4	17	2	39	2.70
**1TMQ**	**E**	**3**	**1**	**3**	**1**	**3**	**1**	**30**
1UDI	E	0	-	0	-	0	-	-
1YVB	E	0	-	1	1	1	2	33.33
**2ABZ**	**E**	**1**	**2**	**1**	**15**	**1**	**2**	**5.56**
**2B42**	**E**	**3**	**1**	**3**	**1**	**3**	**1**	**60**
2J0T	E	0	-	0	-	0	-	-
2MTA	E	0	-	0	-	0	-	0
2O8V	E	0	-	0	-	0	-	-
2OUL	E	0	-	0	-	0	-	-
2PCC	E	0	-	1	1	0	-	0
2SIC	E	0	-	0	-	0	-	-
2SNI	E	0	-	0	-	0	-	0
2UUY	E	0	-	1	9	0	-	0
3SGQ	E	0	-	0	-	0	-	0
4CPA	E	3	51	4	2	3	4	5
7CEI	E	1	5	4	1	2	5	22.22
1AK4	O	0	-	2	1	1	3	33.33
**1AZS**	**O**	**1**	**1**	**1**	**1**	**1**	**1**	**100**
1B6C	O	0	-	0	-	0	-	0
1BUH	O	0	-	0	-	0	-	0
1E96	O	0	-	0	-	0	-	-
1EFN	O	0	-	0	-	0	-	0
1F51	O	0	-	0	-	0	-	0
**1FC2**	**O**	**16**	**1**	**8**	**1**	**20**	**1**	**47.62**
1FCC	O	1	27	0	-	0	-	0

1FFW	O	8	2	3	1	7	2	35
**1FQJ**	**O**	**2**	**1**	**2**	**1**	**4**	**1**	**50**
1GCQ	O	9	1	4	1	7	1	30.43
1GHQ	O	0	-	0	-	0	-	-
1GLA	O	0	-	0	-	0	-	0
**1GPW**	**O**	**1**	**1**	**1**	**1**	**1**	**1**	**33.33**
**1H9D**	**O**	**0**	**-**	**1**	**1**	**1**	**1**	**50**
**1HCF**	**O**	**0**	**-**	**1**	**1**	**1**	**1**	**50**
1HE1	O	0	-	1	1	0	-	0
1J2J	O	0	-	0	-	0	-	0
1JWH	O	0	-	0	-	0	-	-
1KAC	O	4	1	5	1	4	1	40
1KLU	O	0	-	0	-	0	-	-
1KTZ	O	0	-	2	4	0	-	0
**1KXP**	**O**	**2**	**1**	**1**	**1**	**2**	**1**	**66.67**
1ML0	O	0	-	0	-	0	-	0
**1OFU**	**O**	**1**	**1**	**1**	**1**	**1**	**1**	**33.33**
**1PVH**	**O**	**1**	**1**	**0**	**-**	**1**	**1**	**50**
**1QA9**	**O**	**1**	**2**	**1**	**1**	**1**	**1**	**12.50**
1RLB	O	0	-	0	-	0	-	0
1RV6	O	0	-	1	1	1	3	33.33
1S1Q	O	0	-	0	-	0	-	-
1SBB	O	0	-	0	-	0	-	-
**1T6B**	**O**	**3**	**1**	**3**	**1**	**5**	**1**	**55.56**
**1US7**	**O**	**2**	**1**	**2**	**1**	**2**	**1**	**66.67**
1WDW	O	0	-	0	-	0	-	-
1XD3	O	0	-	0	-	0	-	0
1XU1	O	4	85	10	11	3	41	3.45
**1Z0K**	**O**	**0**	**-**	**1**	**1**	**1**	**1**	**33.33**
**1Z5Y**	**O**	**2**	**1**	**2**	**1**	**3**	**1**	**37.50**
**1ZHH**	**O**	**1**	**1**	**1**	**1**	**1**	**1**	**50**
**1ZHI**	**O**	**3**	**1**	**3**	**2**	**3**	**1**	**37.50**
**2A5T**	**O**	**2**	**1**	**1**	**1**	**2**	**1**	**50**
2A9K	O	0	-	2	1	1	2	33.33
2AJF	O	0	-	1	2	0	-	0
**2AYO**	**O**	**2**	**32**	**0**	**-**	**2**	**10**	**3.17**

**2B4J**	**O**	**6**	**1**	**5**	**1**	**8**	**1**	**50**
**2BTF**	**O**	**1**	**6**	**3**	**1**	**1**	**1**	**14.29**
2FJU	O	0	-	0	-	0	-	0
2G77	O	0	-	0	-	0	-	-
2HLE	O	3	2	4	1	3	1	50
**2HQS**	**O**	**7**	**1**	**6**	**1**	**7**	**1**	**38.89**
2OOB	O	0	-	0	-	0	-	-
**2OOR**	**O**	**0**	**-**	**3**	**1**	**3**	**1**	**33.33**
**2VDB**	**O**	**2**	**57**	**1**	**7**	**2**	**18**	**2.90**
**3BP8**	**O**	**3**	**11**	**1**	**28**	**3**	**3**	**7.32**
3D5S	O	0	-	1	4	0	-	0
Medium (26)								
**1BGX**	**A**	**0**	**-**	**1**	**1**	**1**	**1**	**100**
1ACB	E	0	-	0	-	0	-	0
**1JIW**	**E**	**1**	**5**	**0**	**-**	**1**	**5**	**20**
1KKL	E	0	-	0	-	0	-	0
**1M10**	**E**	**2**	**1**	**2**	**1**	**2**	**1**	**50**
1NW9	E	4	1	7	2	4	1	25
**1GRN**	**O**	**6**	**1**	**2**	**1**	**6**	**1**	**66.67**
1HE8	O	9	1	7	1	6	1	40
**1I2M**	**O**	**7**	**1**	**2**	**1**	**7**	**1**	**50**
**1IB1**	**O**	**1**	**1**	**1**	**1**	**1**	**1**	**33.33**
**1K5D**	**O**	**1**	**1**	**1**	**1**	**1**	**1**	**33.33**
1LFD	O	10	1	4	1	9	1	50
**1MQ8**	**O**	**4**	**1**	**0**	**-**	**5**	**1**	**55.56**
**1R6Q**	**O**	**3**	**1**	**2**	**1**	**4**	**1**	**50**
**1SYX**	**O**	**13**	**1**	**8**	**1**	**13**	**1**	**56.52**
1WQ1	O	4	1	2	1	3	1	37.50
1XQS	O	4	1	5	1	4	1	50
1ZM4	O	4	1	2	5	3	2	33.33
**2CFH**	**O**	**9**	**1**	**8**	**1**	**10**	**1**	**76.92**
2H7V	O	5	1	2	1	3	1	42.86
**2HRK**	**O**	**3**	**1**	**2**	**1**	**3**	**1**	**33.33**
**2J7P**	**O**	**1**	**1**	**1**	**1**	**1**	**1**	**50**
2NZ8	O	3	1	1	1	2	1	40
**2OZA**	**O**	**1**	**1**	**1**	**1**	**2**	**1**	**100**

**2Z0E**	**O**	**6**	**1**	**3**	**1**	**8**	**1**	**72.73**
3CPH	O	4	1	5	1	3	1	37.50
Difficult (20)								
**1E4K**	**A**	**1**	**1**	**1**	**1**	**1**	**1**	**50**
1F6M	E	0	-	0	-	0	-	0
**1FQ1**	**E**	**5**	**1**	**1**	**2**	**5**	**1**	**55.56**
1PXV	E	4	1	6	1	4	1	57.14
1ZLI	E	0	-	0	-	0	-	0
**2O3B**	**E**	**1**	**7**	**0**	**-**	**1**	**5**	**12.50**
**1ATN**	**O**	**3**	**1**	**2**	**1**	**3**	**1**	**75**
**1BKD**	**O**	**6**	**1**	**3**	**1**	**8**	**1**	**88.89**
**1FAK**	**O**	**4**	**1**	**1**	**1**	**4**	**1**	**80**
**1H1V**	**O**	**2**	**1**	**1**	**4**	**2**	**1**	**50**
**1IBR**	**O**	**6**	**1**	**2**	**1**	**6**	**1**	**66.67**
**1IRA**	**O**	**3**	**1**	**1**	**1**	**3**	**1**	**42.86**
**1JK9**	**O**	**2**	**1**	**2**	**1**	**3**	**1**	**50**
**1JZD**	**O**	**4**	**1**	**2**	**1**	**4**	**1**	**44.44**
1R8S	O	7	1	2	1	6	1	60
**1Y64**	**O**	**1**	**1**	**1**	**1**	**1**	**1**	**100**
**2C0L**	**O**	**6**	**1**	**3**	**1**	**6**	**1**	**66.67**
**2I9B**	**O**	**3**	**1**	**3**	**1**	**3**	**1**	**50**
2IDO	O	10	1	7	1	9	1	64.29
**2OT3**	**O**	**7**	**1**	**5**	**1**	**7**	**1**	**70**

A/AB: Antigen-unbound/bound Antibody

E: Enzyme-Inhibitor

O: others
