# Supplementary material for: IFACEwat: the interfacial water-implemented re-ranking algorithm to improve the discrimination of near native structures for protein rigid docking
Source: BMC Bioinformatics. 2014 Dec 8;15(Suppl 16):S9. doi: 10.1186/1471-2105-15-S16-S9 (PMC4290663; doi:10.1186/1471-2105-15-S16-S9)

Additional file 2 - Best RMS of conformations in the top 10, 100, and 1000 ranks found by the IFACEwat against ZDOCK (*left*) and ZRANK (*right*). Each dot represents a protein complex case according to difficulty level: easy (black), medium (red), and difficult (blue).

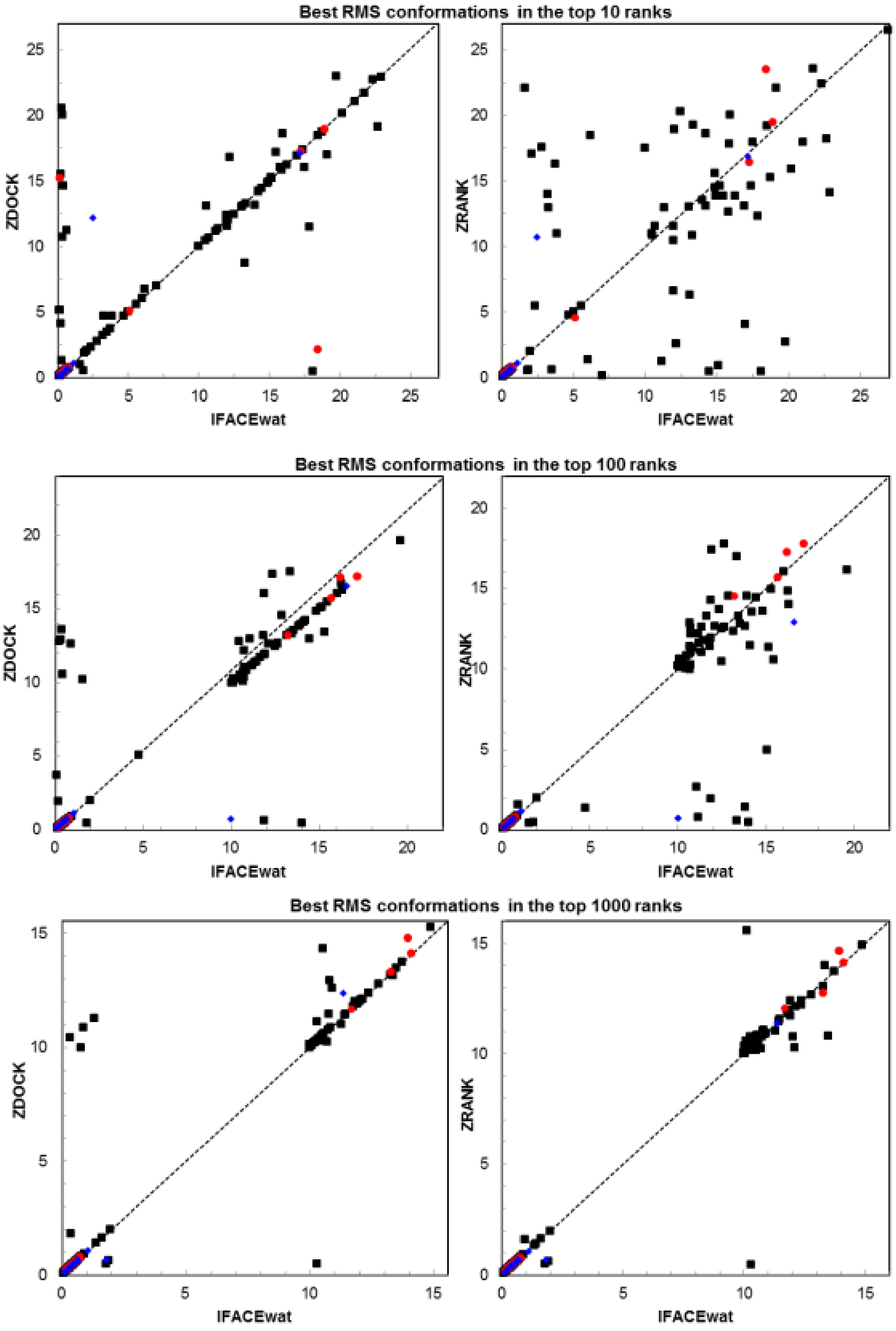

Supplement: Additional file 2 — Best RMS of conformations in the top 10, 100, and 1000 ranks found by the IFACEwat against ZDOCK (left) and ZRANK (right). Each dot represents a protein complex case according to difficulty level: easy (black), medium (red), and difficult (blue). [file 1471-2105-15-S16-S9-S2.pdf]
