# Supplementary material for: IFACEwat: the interfacial water-implemented re-ranking algorithm to improve the discrimination of near native structures for protein rigid docking
Source: BMC Bioinformatics. 2014 Dec 8;15(Suppl 16):S9. doi: 10.1186/1471-2105-15-S16-S9 (PMC4290663; doi:10.1186/1471-2105-15-S16-S9)
Supplement: Additional file 3 — Re-rankings results that are equivalent with or better than the F2Dock-GB rerank. To maintain a rational assessment, results between each of the methods and ZDOCK3.0.2 separately are used as the intermediate comparison since all the 3 methods are applied for the same dataset of protein complexes (the benchmark 4.0) with 15o rotational sampling. Results of F2Dock are done by Chowdhury et al. Filename: AdditionalFile-3.pdf [file 1471-2105-15-S16-S9-S3.pdf]

**Additional file 3 – Re-rankings results that are equivalent with or better than the F$^2$Dock.** To maintain a rational assessment, results between each of the methods and ZDOCK separately are used as the intermediate comparison since all the 3 methods are applied for the same dataset of protein complexes (the benchmark 4.0) with 15$^o$ rotational sampling. Results of F$^2$Dock are done by Chowdhury et al.

| Com-plex | Type | Rank of the first Hit | | | | RMS of the first Hit | | | |
|---|---|---|---|---|---|---|---|---|---|
| | | ZDOCK | IFACEwat | ZDOCK | F$^2$Dock | ZDOCK | IFACEwat | ZDOCK | F$^2$Dock |
| *Easy* | | | | | | | | | |
| 1BVK | A | 1 | 2 | 184 | 205 | 0.94 | 1.61 | 3.6 | 4.9 |
| 1DQJ | A | 1 | 1 | 374 | 74 | 0.23 | 1.56 | 4.0 | 4.9 |
| 1E6J | A | 1 | 2 | 3 | 126 | 0.42 | 0.42 | 4.1 | 5 |
| 1JPS | A | 1 | 1 | 1266 | 186 | 0.3 | 0.3 | 2.1 | 2.7 |
| 1MLC | A | 1 | 1 | 57 | 11 | 0.48 | 0.48 | 2 | 3.8 |
| 1WEJ | A | 1 | 1 | 9 | 5 | 0.5 | 0.5 | 1.5 | 3.2 |
| 2FD6 | A | 1 | 1 | 3 | 62 | 0.55 | 0.55 | 5.0 | 4.4 |
| 2I25 | A | 1 | 1 | 2 | 122 | 0.6 | 0.6 | 3.0 | 3.9 |
| 2VIS | A | 1 | 3 | - | - | 0.68 | 0.68 | - | - |
| 1IQD | AB | - | 1 | 18 | 4 | - | 0.35 | 4.3 | 3.0 |
| 1K4C | AB | 1 | 1 | 583 | 105 | 0.49 | 0.49 | 2.9 | 4.4 |
| 1KXQ | AB | - | 1 | 2 | 1 | - | 0.09 | 1.2 | 1.6 |
| 1NSN | AB | - | - | 1267 | - | - | - | 1.6 | - |
| 1AVX | E | 1 | 1 | 25 | 1 | 0.31 | 0.31 | 3.5 | 4.5 |
| 1BVN | E | 1 | 1 | 3 | 1 | 0.21 | 0.21 | 1.2 | 3.2 |
| 1CLV | E | 1 | 1 | 3 | 1 | 0.35 | 0.35 | 2.3 | 2.5 |
| 1DFJ | E | 1 | 1 | 1 | 9 | 0.47 | 0.47 | 4.1 | 4.4 |
| 1E6E | E | 1 | 1 | 5 | 20 | 0.11 | 0.11 | 3.2 | 4.7 |
| 1GXD | E | 1 | 1 | 1173 | - | 0.8 | 0.8 | 4.9 | - |
| 1JTG | E | 1 | 1 | 1 | 7 | 0.19 | 0.19 | 2.6 | 4.6 |
| 1OC0 | E | - | - | 1590 | - | - | - | 4.8 | - |
| 1OPH | E | 1 | 1 | 1694 | - | 0.49 | 1.81 | 3.9 | - |
| 1TMQ | E | 1 | 1 | 16 | 1 | 0.33 | 0.33 | 3.6 | 4.8 |
| 1YVB | E | - | 2 | 1 | - | - | 1.86 | 2.4 | - |
| 2ABZ | E | 2 | 2 | - | 5 | 2.25 | 2.25 | - | 2.8 |
| 2B42 | E | 1 | 1 | 3 | 1 | 0.24 | 0.24 | 4.2 | 3.9 |
| 1AK4 | O | - | 3 | 1090 | 964 | - | 0.36 | 3.5 | 4.3 |
| 1AZS | O | 1 | 1 | 42 | - | 1.99 | 1.99 | 2.9 | - |
| 1EFN | O | - | - | - | - | - | - | - | - |

| | | | | | | | | | |
|---|---|---|---|---|---|---|---|---|---|
| 1F51 | O | - | - | 589 | - | - | - | 4.6 | - |
| 1FC2 | O | 1 | 1 | - | 1190 | 0.44 | 0.44 | - | 5.0 |
| 1FCC | O | 27 | - | - | - | 8.33 | - | - | - |
| 1FFW | O | 2 | 2 | 73 | 325 | 2.56 | 0.35 | 4.5 | 4.7 |
| 1FQJ | O | 1 | 1 | - | - | 0.31 | 0.31 | - | - |
| 1GCQ | O | 1 | 1 | 1105 | - | 0.42 | 0.42 | 1.4 | - |
| 1GHQ | O | - | - | - | - | - | - | - | - |
| 1GLA | O | - | - | 1708 | - | - | - | 3.9 | - |
| 1GPW | O | 1 | 1 | 3 | 1 | 0.48 | 0.48 | 3.6 | 3.7 |
| 1H9D | O | - | 1 | 1006 | - | - | 0.63 | 4.5 | - |
| 1HCF | O | - | 1 | 175 | 1225 | - | 0.29 | 4.0 | 4.7 |
| 1JWH | O | - | - | 7 | - | - | - | 3.6 | - |
| 1KAC | O | 1 | 1 | 592 | 8 | 0.32 | 0.32 | 4.5 | 4.4 |
| 1KLU | O | - | - | 1957 | - | - | - | 3.4 | - |
| 1KXP | O | 1 | 1 | 1 | 7 | 0.57 | 0.57 | 1.6 | 3.5 |
| 1OFU | O | 1 | 1 | 84 | - | 0.45 | 0.45 | 4.5 | - |
| 1PVH | O | 1 | 1 | 748 | - | 2.08 | 2.08 | 4.5 | - |
| 1QA9 | O | 2 | 1 | - | - | 0.13 | 0.13 | - | - |
| 1S1Q | O | - | - | 756 | - | - | - | 1.9 | - |
| 1SBB | O | - | - | - | - | - | - | - | |
| 1T6B | O | 1 | 1 | 58 | 525 | 0.15 | 0.15 | 3.6 | 4.0 |
| 1US7 | O | 1 | 1 | 74 | - | 0.48 | 0.48 | 1.1 | - |
| 1XU1 | O | 85 | 41 | 912 | - | 2.01 | 2.01 | 5.0 | - |
| 1Z0K | O | - | 1 | 8 | 307 | - | 0.22 | 3.3 | 3.3 |
| 1Z5Y | O | 1 | 1 | 20 | - | 0.31 | 0.31 | 3.4 | - |
| 1ZHH | O | 1 | 1 | - | - | 0.43 | 0.43 | - | - |
| 1ZHI | O | 1 | 1 | 65 | 202 | 0.23 | 0.23 | 4.4 | 4.0 |
| 2A5T | O | 1 | 1 | - | 268 | 0.73 | 0.73 | - | 3.6 |
| 2A9K | O | - | 2 | - | 558 | - | 0.41 | - | 3.4 |
| 2AJF | O | - | - | 475 | - | - | - | 3.6 | - |
| 2AYO | O | 32 | 10 | 37 | 1108 | 2.06 | 2.31 | 3.3 | 2.0 |
| 2B4J | O | 1 | 1 | - | - | 0.54 | 0.54 | - | - |
| 2BTF | O | 6 | 1 | 53 | 95 | 0.45 | 0.45 | 4.7 | 4.5 |
| 2HLE | O | 2 | 1 | 31 | 4 | 2.16 | 0.44 | 4.1 | 3.8 |
| 2HQS | O | 1 | 1 | - | 27 | 0.55 | 0.55 | - | 4.1 |
| 2OOB | O | - | - | - | - | - | - | - | - |

| | | | | | | | | | |
|---|---|---|---|---|---|---|---|---|---|
| 2OOR | O | - | 1 | 766 | 16 | - | 0.21 | 4.4 | 4.0 |
| 2VDB | O | 57 | 18 | 5 | - | 2.06 | 2.36 | 1.2 | - |
| 3BP8 | O | 11 | 3 | - | 474 | 2.69 | 2.46 | - | 5.0 |
| *Medium* | | | | | | | | | |
| 1BGX | A | - | 1 | - | - | - | 0.17 | - | - |
| 1JIW | E | 5 | 5 | - | - | 5.21 | 2.15 | - | - |
| 1KKL | E | - | - | - | - | - | - | - | - |
| 1M10 | E | 1 | 1 | - | - | 0.78 | 0.78 | - | - |
| 1NW9 | E | 1 | 1 | - | 321 | 0.76 | 0.76 | - | 4.9 |
| 1GRN | O | 1 | 1 | 1299 | 401 | 0.52 | 0.52 | 4.3 | 4.8 |
| 1HE8 | O | 1 | 1 | - | - | 0.31 | 0.31 | - | - |
| 1I2M | O | 1 | 1 | 267 | 545 | 0.56 | 0.56 | 2.2 | 2.6 |
| 1IB1 | O | 1 | 1 | - | - | 0.51 | 0.51 | - | - |
| 1K5D | O | 1 | 1 | - | 521 | 0.35 | 0.35 | - | 4.3 |
| 1LFD | O | 1 | 1 | 85 | 990 | 0.24 | 0.24 | 4.6 | 4.6 |
| 1MQ8 | O | 1 | 1 | 1455 | - | 0.27 | 0.27 | 3.2 | - |
| 1R6Q | O | 1 | 1 | - | 180 | 0.28 | 0.28 | - | 3.7 |
| 1SYX | O | 1 | 1 | 211 | 2 | 0.56 | 0.56 | 4.8 | 4.7 |
| 1WQ1 | O | 1 | 1 | 81 | - | 0.29 | 0.29 | 4.0 | - |
| 1XQS | O | 1 | 1 | 19 | 61 | 0.44 | 0.44 | 3.8 | 4.2 |
| 1ZM4 | O | 1 | 2 | 6 | - | 0.33 | 0.33 | 4.1 | - |
| 2CFH | O | 1 | 1 | 1 | 119 | 0.59 | 0.59 | 3.8 | 2.6 |
| 2H7V | O | 1 | 1 | 1112 | - | 0.44 | 0.44 | 4.6 | - |
| 2HRK | O | 1 | 1 | 3 | - | 0.39 | 0.39 | 3.7 | - |
| 2J7P | O | 1 | 1 | - | - | 0.53 | 0.53 | - | - |
| 2NZ8 | O | 1 | 1 | 64 | - | 0.35 | 0.35 | 4.5 | - |
| 2OZA | O | 1 | 1 | - | - | 0.53 | 0.53 | - | - |
| 2Z0E | O | 1 | 1 | - | 169 | 0.61 | 0.61 | - | 3.9 |
| 3CPH | O | 1 | 1 | - | 250 | 0.44 | 0.44 | - | 4.3 |
| *Difficult* | | | | | | | | | |
| 1E4K | A | 1 | 1 | - | - | 0.27 | 0.27 | - | - |
| 1F6M | E | - | - | - | - | - | - | - | - |
| 1FQ1 | E | 1 | 1 | - | - | 0.68 | 0.68 | - | - |
| 1PXV | E | 1 | 1 | - | - | 0.12 | 0.12 | - | - |
| 1ZLI | E | - | - | - | - | - | - | - | - |
| 2O3B | E | 7 | 5 | - | - | 2.47 | 2.47 | - | - |

| | | | | | | | | | |
|---|---|---|---|---|---|---|---|---|---|
| 1ATN | O | 1 | 1 | - | 1307 | 0.26 | 0.26 | - | 2.7 |
| 1BKD | O | 1 | 1 | - | - | 0.45 | 0.45 | - | - |
| 1FAK | O | 1 | 1 | - | - | 0.17 | 0.17 | - | - |
| 1H1V | O | 1 | 1 | - | - | 0.59 | 0.59 | - | - |
| 1IBR | O | 1 | 1 | - | - | 0.32 | 0.32 | - | - |
| 1IRA | O | 1 | 1 | - | - | 0.65 | 0.65 | - | - |
| 1JK9 | O | 1 | 1 | 510 | 422 | 0.15 | 0.15 | 4.2 | 2.5 |
| 1JZD | O | 1 | 1 | 44 | 144 | 0.53 | 0.53 | 4.6 | 4 |
| 1R8S | O | 1 | 1 | - | - | 0.47 | 0.47 | - | - |
| 1Y64 | O | 1 | 1 | - | - | 1.08 | 1.08 | - | - |
| 2C0L | O | 1 | 1 | - | - | 0.46 | 0.46 | - | - |
| 2I9B | O | 1 | 1 | - | - | 0.65 | 0.65 | - | - |
| 2IDO | O | 1 | 1 | 130 | 156 | 0.38 | 0.38 | 3.6 | 4.5 |
| 2OT3 | O | 1 | 1 | 121 | - | 0.6 | 0.6 | 4.6 | - |
