# Supplementary material for: IFACEwat: the interfacial water-implemented re-ranking algorithm to improve the discrimination of near native structures for protein rigid docking
Source: BMC Bioinformatics. 2014 Dec 8;15(Suppl 16):S9. doi: 10.1186/1471-2105-15-S16-S9 (PMC4290663; doi:10.1186/1471-2105-15-S16-S9)

Additional file 4 - Angle and distance constraints to define a water-mediated Hydrogen bond according to the solvated rotamer library referred from Jiang *et al.* *d* is the distance between the water (W) oxygen and polar (acceptor A/donor D) atoms. H and AB are Hydrogen and Acceptor-Base atoms respectively.

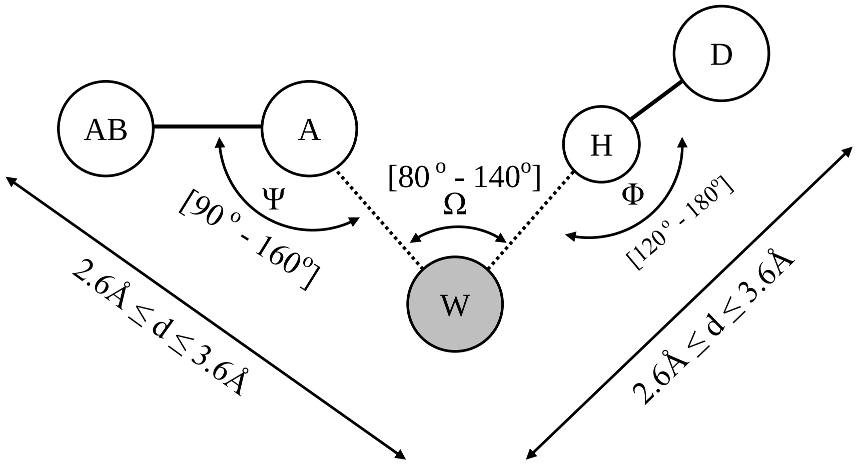

Supplement: Additional file 4 — Angle and distance constraints to define a water-mediated Hydrogen bond according to the solvated rotamer library by Jiang et al. d is the distance between the water (W) oxygen and polar (acceptor A/donor D) atoms. H and AB are Hydrogen and Acceptor-Base atoms respectively. [file 1471-2105-15-S16-S9-S4.pdf]
